# The farnesoid X receptor activates transcription independently of RXR at non-canonical response elements

**DOI:** 10.1093/nar/gkae1214

**Published:** 2024-12-09

**Authors:** Suzanne W van der Veen, Jelmer J Dijkstra, Ellen C L Willemsen, René Houtman, Alexandra Milona, Nikolas Marchet, Maureen Spit, Danielle Hollman, Fried J T Zwartkruis, Michiel Vermeulen, Jose M Ramos Pittol, Saskia W C van Mil

**Affiliations:** Center for Molecular Medicine, University Medical Center Utrecht, Utrecht University, Stratenum Building, Universiteitsweg 100, 3584CG Utrecht, The Netherlands; Department of Molecular Biology, Faculty of Science, Radboud Institute for Molecular Life Sciences, Oncode Institute, Radboud University Nijmegen, Geert Grooteplein 26-28, 6525GA Nijmegen, The Netherlands; Division of Molecular Genetics, The Netherlands Cancer Institute, Plesmanlaan 121, 1066CX Amsterdam, The Netherlands; Center for Molecular Medicine, University Medical Center Utrecht, Utrecht University, Stratenum Building, Universiteitsweg 100, 3584CG Utrecht, The Netherlands; Precision Medicine Lab, Antoni van Leeuwenhoek Building, Kloosterstraat 9, 5349AE Oss, The Netherlands; Center for Molecular Medicine, University Medical Center Utrecht, Utrecht University, Stratenum Building, Universiteitsweg 100, 3584CG Utrecht, The Netherlands; The Francis Crick Institute, 1 Midland Road, London NW11AT, UK; Institute of Biochemistry, University of Innsbruck, Innrain 80-82, Innsbruck, Tirol A-6020, Austria; Center for Molecular Medicine, University Medical Center Utrecht, Utrecht University, Stratenum Building, Universiteitsweg 100, 3584CG Utrecht, The Netherlands; Center for Molecular Medicine, University Medical Center Utrecht, Utrecht University, Stratenum Building, Universiteitsweg 100, 3584CG Utrecht, The Netherlands; Center for Molecular Medicine, University Medical Center Utrecht, Utrecht University, Stratenum Building, Universiteitsweg 100, 3584CG Utrecht, The Netherlands; Department of Molecular Biology, Faculty of Science, Radboud Institute for Molecular Life Sciences, Oncode Institute, Radboud University Nijmegen, Geert Grooteplein 26-28, 6525GA Nijmegen, The Netherlands; Division of Molecular Genetics, The Netherlands Cancer Institute, Plesmanlaan 121, 1066CX Amsterdam, The Netherlands; Institute of Biochemistry, University of Innsbruck, Innrain 80-82, Innsbruck, Tirol A-6020, Austria; Center for Molecular Medicine, University Medical Center Utrecht, Utrecht University, Stratenum Building, Universiteitsweg 100, 3584CG Utrecht, The Netherlands

## Abstract

The farnesoid X receptor (FXR) is a nuclear receptor (NR) known to obligately heterodimerize with the retinoid X receptor (RXR). FXR is expressed as four isoforms (α1–α4) that drive transcription from IR-1 (inverted repeat-1) response elements (REs). Recently, we found that FXR isoforms α2/α4 also activate transcription from non-canonical ER-2 (everted repeat-2) REs, mediating most metabolic effects of general FXR activation. Here, we explored molecular determinants of regulation by FXRα2 from ER-2 REs through quantitative interaction proteomics, site-directed mutagenesis and transcriptomics. We discovered FXRα2 binds to and activates ER-2 elements *in vitro* and in reporter assays independently of RXR. Genome-wide binding analysis in mouse liver revealed higher ER-2 motif enrichment in FXR sites lacking RXR. Abrogation of FXRα2:RXR heterodimerization abolished IR-1, but preserved ER-2 transactivation. Transcriptome-wide, RXR overexpression inhibited 25% of FXRα2 targets in HepG2. These genes were specifically activated by the heterodimerization-deficient mutant FXRα2^L434R^, enriched for ER-2 motifs at their promoters, and involved in lipid metabolism and ammonia detoxification. In conclusion, RXR acts as a molecular switch, inhibiting FXRα2 activation from ER-2 while enhancing it from canonical IR-1 REs. Our results showcase FXR as the first NR with isoform-specific RXR-independent REs, highlighting a new layer of regulation and complexity for RXR-heterodimerizing NRs.

## Graphical Abstract

**Graphical Abstract:**
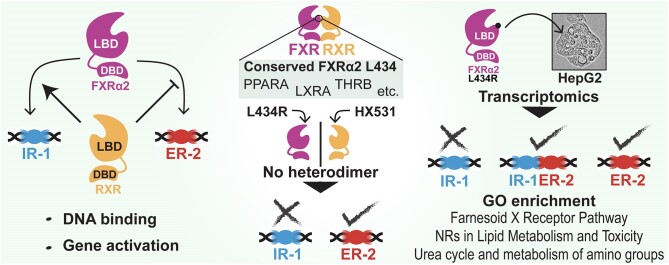

## Introduction

The farnesoid X receptor (FXR; encoded by the *NR1H4* gene) is a nuclear receptor (NR) activated by bile acids that is highly expressed in the liver, intestine, kidney, and adrenal glands. Treatment with ligands of FXR (e.g. obeticholic acid, OCA) results in hepatic lipid clearance and reduction of fibrosis and inflammation in mouse models of steatotic liver disease [SLD; reviewed by Panzitt *et al.* (1)]. However, the response in patients tends to be heterogeneous, and side-effects reduce the quality of life (2). Therefore, there is a pressing need to understand the molecular mechanisms related to FXR activation, to optimize current therapies.

FXR is a member of the class II NRs, considered obligate heterodimerization partners of the retinoid X receptor (RXR) (3,4). FXR is expressed as four isoforms (FXRα1–FXRα4) from the same genomic locus via differential promoter usage and alternative splicing (Supplementary Figure S1A). As a heterodimer with RXR, all FXR isoforms regulate transcription by binding to FXR response elements (REs) containing inverted repeats-1 (IR-1; inverted hexameric repeat spaced by one nucleotide) DNA motifs. We have recently shown that FXRα2 and FXRα4 additionally bind to everted repeats-2 (ER-2; everted hexameric repeat spaced by two nucleotides) DNA motifs, and show enhanced gene activation from overlapping ER-2/IR-1 motifs (5). The ER-2 motif was present in the majority of FXRα2-specific binding sites in mouse liver organoids and was required for most OCA-driven changes in metabolic gene expression (5). Modulating the DNA binding selectivity of FXRα2 could potentially be used to enhance the beneficial effects of FXR agonism in targeting metabolic diseases. Therefore, we aimed to characterize the regulation of FXRα2 binding to the non-canonical ER-2 DNA motifs in liver cells.

We discovered that RXR, the canonical heterodimerization partner of FXR, is not required for FXRα2 binding to ER-2. Instead, RXR inhibits FXRα2 transactivation from ER-2 sites in luciferase reporters and in HepG2 cells. We generated an RXR heterodimerization-deficient FXRα2 mutant that retained ER-2-binding capacity. This mutant was able to activate genes involved in lipid metabolism and ammonia disposal upon agonist treatment, while presenting fewer repressive effects than wt FXRα2. Our results reveal the potential of inhibiting FXR heterodimerization as a strategy to tailor the response to FXR agonism in liver cells.

## Materials and methods

### Reagents

Polyclonal antibodies against FXR and RXRα (Santa Cruz Biotechnology, Dallas, TX; sc-13063 and sc-553, respectively) were used for electrophoretic mobility shift assay (EMSA) supershift assays and chromatin immunoprecipitation (ChIP) experiments. Antibodies against β-actin (Santa Cruz Biotechnologies, Dallas, TX, sc47778), FXR (Invitrogen, Carlsbad, CA; 417 200), RXRα (Santa Cruz Biotechnology, Dallas, TX; sc-553), and FLAG-tag (Sigma-Aldrich; F1804) were used for western blot. Obeticholic acid was kindly provided by Luciano Adorini (previously Intercept Pharmaceuticals, Inc.) and was diluted from a 10 mM stock solution in dimethylsulfoxide (DMSO). HX531 (Sigma-Aldrich, SML2170) was diluted from a 4 mM stock solution in DMSO. Protein A agarose beads were purchased from Roche, and Di(*N*-succinimidyl) glutarate (DSG), 97% from Synchem UG.

### Biological resources

*Plasmids*. Coding sequences (CDS) from human FXR isoforms α1 (NM_001206979.2), α2 (NM_005123.4), RXRα (NM_002957), RXRβ (NM_021976.5) and PPARα (NM_001362872) were amplified from human complementary DNA. All CDS were amplified by polymerase chain reaction (PCR) and cloned into vectors containing a MYC tag followed by a TwinStrep tag, MYC tag followed by a VP16 transactivation domain (TAD) (Noted as -VP16), or FLAG tag at the C-terminus. FXRα2^L434R^ and FXRα2^L415R^ were generated by site-directed mutagenesis using QuikChange II Site-directed mutagenesis kit (Agilent, primers can be found in Supplementary Table S1). pCDNA3.1 (Invitrogen) vectors were used for transient overexpression, luciferase reporter assays and *in vitro* translations, pLV(hEF1α)-IRES-NeoR and pCW-57-GFP-2A-MCS derived vectors were used for stable transductions. Luciferase reporter plasmids containing IR-1, ER-2 and IR-1/ER-2 series motifs were based on pGL3 vector containing Luc2 under the control of a minimal promoter (Promega, Madison, WI) as described previously (5). Briefly, motif sequences derived from FXR peaks at the mouse *Nr0b2* and *Osgin1* loci (IR-1), the *Ass1* and *Mpc1* loci (ER-2) and the *Nr0b2* enhancer (ER-2/IR-1) were cloned as individual motifs at the promoter and enhancer portions of the pGL3 vector at the XhoI and SalI sites. Primer sequences and genomic coordinates can be found in Supplementary Table S2. Split luciferase backbone vectors pBIT2.1-N-[TK-SMBIT] and pBIT1.1-N-[TK-LGBIT] and control vectors pBIT2.1-N-LgBIT-PRKAR2A and pBIT2.1-N-SmBIT-PRKACA were part of the NanoBiT PPI Starter System (Promega). pCW57-GFP-2A-MCS was a gift from Adam Karpf (Addgene plasmid # 71 783; http://n2t.net/addgene:71783;RRID:Addgene_71 783) (6).

*Mice*. Studies were approved by the University Medical Center Utrecht ethics committee and conducted in accordance with European law. 9–12-week-old male C57BL/6 mice were housed in a room with controlled temperature (20–24°C), relative humidity (55% ± 15%), and a 12-h light/dark cycle. Mice were fed chow and water ad libitum. On the last day, the food was removed in the morning, and livers were harvested 4 h afterwards.

*Cell culture*. HEK-293T were grown in Dulbecco’s modified Eagle medium (DMEM) (Lonza, Basel, Switzerland 12–604F) supplemented with 10% fetal bovine serum (FBS) (Bodinco BV, Alkmaar, Netherlands) and 1% Penicillin/Streptomycin (Sigma-Aldrich, P0781). HepG2 cells were grown in DMEM (Lonza, 12–707F) supplemented with 10% FBS and 1% L-glutamine (Lonza, 17–605E).

*Lentiviral transduction and drug treatments of HepG2 cells*. HEK-293T cells were co-transfected with 3^rd^ generation lentiviral packaging (Sigma-Aldrich) and pCW57-tGFP-based plasmid as such (GFP) or containing either FXRα2^wt^ or FXRα2^L434R^. A total of 48 h after transfection, HepG2 cells were transduced with the HEK-293T supernatant containing lentiviral particles. After selection using 2 μg/ml puromycin (Sigma-Aldrich, P8833), the procedure was repeated using pLV(hEF1α) vectors expressing RXRα-FLAG or H2B-Neon as control. Afterwards, the cells were selected with 2 mg/ml G418 (Thermo Fisher, 11811031). Prior to every FXR stimulation experiment, transduced HepG2 cells were treated with 2 μg/ml doxycycline hydrochloride (D3447, Sigma-Aldrich) for 48 h in full culture media. Afterwards, medium was exchanged to DMEM supplemented with 1% FBS, doxycycline and L-glutamine, containing either 10 μM OCA in DMSO or 0.1% DMSO as vehicle treatment. Cells were lysed for RNA extraction after overnight treatment. To test the effect of the RXR inhibitor HX531 on FXR gene activation, HepG2 cells transduced with doxycycline-inducible FXRα2^wt^ and constitutive RXRα were used. After induction of FXRα2^wt^, medium was exchanged to DMEM supplemented with 1% FBS, doxycycline, and L-glutamine containing either 10 μM OCA in DMSO, or 0.1% DMSO as vehicle treatment, in the presence or absence of 4 μM HX531. Cells were lysed for RNA extraction after 8 h of treatment.

### Electrophoretic mobility shift assays

FXRα2, FXRα2^L434R^, RXRα, RXRβ and GFP proteins were *in vitro* translated from pCDNA3.1 vectors using the TNT Quick Coupled Transcription/Translation system (Promega). Oligonucleotide probes containing ER-2, IR-1 or ER-2/IR-1 sequences (IDT) were labelled with EasyTides γ-^32^P ATP, 3000Ci/mmol (Revvity) using T4-PNK (NEB). Sequences analysed were CCTGGGTTAATGACCCTGTT for IR-1, based on the mouse *Nr0b2/Shp* promoter, and AAGTGACCGAGCGGACAGCG for ER-2, based on the mouse *Mpc1* promoter (5). For the analysis of ER-2/IR-1, a series of probes based on the sequence GACTGCCCGAGAGGTCAGCGACCTGCC (ER-2/IR-1) (5) and sequences mutagenized to inactivate specific NR-hexamers were used. Probe sequences can be found in Supplementary Table S3. Binding reactions contained 20 mM 4-(2-hydroxyethyl)-1-piperazineethanesulfonic acid (HEPES), pH 7.4, 10% glycerol, 50 mM KCl, 1 mM Dithiothreitol (DTT), 10 μg bovine serum albumin (BSA) and 1 μg of poly-dIdC. A total of 1 μl *in vitro* translated FXR and 0.2 μl RXR were used unless a titration is declared. For titration experiments, 2 μl of the indicated *in vitro* translated protein was used as the highest concentration, followed by sequential 1:2 dilutions. Samples were pre-incubated at room temperature for 10 min prior to the addition of ^32^P-labeled double-stranded oligonucleotide probes as indicated. In supershift experiments, 1 μg of antibodies targeting FXR or RXR, or normal rabbit IgG were added. Where indicated, cold probes were added to the pre-incubation mix at a 12-fold molar excess. Cold probe competition was performed using either wild type sequences, or mutants in which only one of the two NR-hexamers was inactivated (Supplementary Table S3). Samples were kept at room temperature for further 30 min, followed by 30 min at 4°C. The protein-DNA complexes were resolved on a pre-electrophoresed 4% polyacrylamide gel in 0.5× 'TBE buffer' (44.5 mM Tris base, 44.5 mM Sodium Borate and 1 mM EDTA). ^32^P-labelled probes were detected by autoradiography using a Typhoon FLA 9500 scanner (GE).

### ChIP followed by sequencing

A total of 30 mg of snap-frozen liver tissue from a wt mouse was crosslinked using DSG and formaldehyde, as described in Bijsmans *et al.* (7). The nuclei were extracted and sonicated to yield 500–1000 base pair (bp) DNA fragments. ChIP was performed according to Ramos-Pittol *et al.* using rabbit polyclonal antibodies against FXR or RXRα (8). Purified DNA from immunoprecipitated material and input DNA from each sample were re-sonicated in a Covaris sonicator to achieve an average fragment size of around 300 bp. Sequencing libraries were prepared using the SOLiD ChIP-seq kit and sequenced on a SOLiD 5500 WF platform. Alignment to the mm10 reference genome was carried out using Bowtie2 allowing for up to two mismatches (9). FXR and RXRα peaks were identified using MACS2 (10) with parameters ‘–nomodel –extsize 150’, and additionally ‘-q 0.1’ for RXRα (Supplementary Data SD2). Genomic distances were calculated using BEDtools, and FXR peaks located within 1 kb upstream or downstream of RXRα peaks were considered proximal. HOMER findmotifsgenome was used to determine DNA motif enrichment in genomic regions (11). Motifs for the ER-2 and ER-2/IR-1 were generated *de novo* using HOMER findmotifsgenome from FXRα2 ChIP-seq datasets under accession GSE133700 (5) with argument ‘-len 14,20’ and were included in the vertebrate Known Motif list of HOMER prior to analysis (Supplementary Data SD3) (11). Coverage was computed with DeepTools2 bamCoverage with options ‘–binsize 50 –normalizeUsing RPGC –effectiveGenomeSize 1 865 500 000 –extendReads 300’ (12). Images of TF occupancy at individual loci were generated with the UCSC genome browser (http://genome-euro.ucsc.edu/) and coverage is expressed as reads per genomic content (12). Genomic coordinates shown can be found in Supplementary Table S4. ChIP-seq data generated in this study are publicly available at the gene expression omnibus (25) under accession number GSE73624.

### Nuclear extraction for co-immunoprecipitation and DNA pulldown

HEK293T cells were seeded in 10 cm dishes and transfected with 10 μg pCDNA3.1-FXRα2-MycTwinstrep, pCDNA3.1-FXRα2^L434R^-MycTwinstrep, pCDNA3.1-FXRα2^L415R^-MycTwinstrep or pCDNA3.1-GFP and 1 μg pCDNA3.1-RXRα-FLAG using *Trans*IT-LT1 Transfection reagent (Mirus, MIR 2304). A total of 48 h after transfection, nuclear extracts for co-immunoprecipitation (co-IP) were prepared. HepG2 cells overexpressing FXRα2^wt^ were cultured in medium with FBS replaced with dialyzed FBS (Gibco) 24h before harvesting, and cells were treated with 10 μM OCA for 1 h before harvesting. Afterwards, nuclear lysates were prepared as described previously (13). Briefly, cells were incubated in hypotonic Buffer A [10 mM HEPES (pH 7.9), 1.5 mM MgCl_2_, 10 mM KCl and 0.15% NP40], after which they were lysed by dounce homogenization. Nuclei were collected by centrifugation at 4°C and lysed in Buffer C [420 mM NaCl, 20 mM HEPES pH 7.9, 20% (*v/v*) glycerol, 2 mM MgCl_2_, 0.2 mM ethylenediaminetetraacetic acid (EDTA), 0.1% NP40, EDTA-free complete protease inhibitors (Roche) and 0.5 mM DTT] for 1 h at 4°C. The soluble fraction was collected as nuclear extract and used directly for co-IP, or snap frozen in liquid nitrogen and stored at −80°C until further use.

### Co-immunoprecipitation

The NaCl concentration of the nuclear extracts was set to 0.1 M with hypotonic buffer, and 1 μg rabbit α-RXRα (Santa Cruz Biotechnology, sc-553) was added. After overnight rotation at 4°C, 30 μl of G-Sepharose beads (Thermo Fisher, 20 397) were added and the mix was incubated for 1 h at 4°C. The beads were washed four times with 800 μl wash buffer (150 mM NaCl, 50 mM Tris-HCl, pH 8.0, 1 mM EDTA, 0.1% NP40, 10% glycerol, 1 mM DTT and EDTA-free complete protease inhibitors). A total of 50 μl Laemmli sample buffer was added followed by 5 min at 95°C and the soluble fraction was collected. Samples were further analysed by western blot.

### Western blotting

Total cell lysates were prepared using RIPA (25 mM Tris-HCl, pH 8.0, 1% Triton X-100, 0.5% sodium deoxycholate, 25 mM EDTA, 150 mM NaCl, 0.1% sodium dodecyl sulfate and EDTA-free complete protease inhibitors) and incubated on ice for 20 min, prior to centrifugation. After centrifugation, the soluble fraction was collected. Laemmli sample buffer was added followed by 5 min incubation at 95°C. Samples were loaded on 10% sodium dodecyl sulphate-polyacrylamide gel electrophoresis gel and transferred to a polyvinylidene difluoride (PVDF) membrane. The membranes were probed overnight at 4°C using antibodies targeting FXR (1:1000), FLAG-tag (1:5000) or RXRα (1:1000), and subsequently incubated with suitable Horseradish peroxidase (HRP)-conjugated secondary antibodies (Dako). Mouse β-actin-HRP antibody (1:5000) was used for the loading control. Signals were detected with enhanced chemiluminescence using LAS4000 (GE).

### DNA pulldown

DNA oligomers were purchased with a 5'-azidogroup attached to the forward strand (IDT, sequences can be found in Supplementary Table S5). Oligos were annealed by combining 500 pmol of the forward and 750 pmol of the reverse strand in 2× annealing buffer (20 mM Tris, pH 8.0, 100 mM NaCl), denatured for 10 min at 95°C and left to cool overnight for hybridization. The oligos were bound to alkyne-beads using a click-reaction using the contents of the Click-iT protein Enrichment Kit (Invitrogen) by combining 1 μmol oligos in 50 μl annealing buffer with 50 μl bead slurry, 200 μl DNA binding buffer (DBB: 1 M NaCl, 0.05% NP40, 10 mM Tris, pH 8.0) and 250 μl Click-iT 2× catalyst solution (manufacturer’s instructions). The mixture was incubated for 18 h at room temperature (RT) in the dark while rotating, followed by washing once with DBB and twice with protein binding buffer (PBB: 50 mM NaCl, 0.25% NP40, 50 mM Tris, pH 8.0, EDTA-free complete protease inhibitors and 1 mM DTT). The beads were then split in two, which were used for the forward and reverse pull down reactions. Subsequent steps were performed at 4°C. Nuclear extract (500 μg) and 10 μg of competitor DNA (5 μg poly-dIdC, 5 μg poly-dAdT) were added to PBB to 600 μl total. Proteins were allowed to bind to the oligos for 90 min while rotating. The beads were washed three times with PBB and two times with PBS. The beads were resuspended in freshly prepared elution buffer (2 M urea, 100 mM Tris, pH 8.0, 10 mM DTT in ultra-pure H_2_O) and incubated for 20 min at RT while shaking at 1200 rpm. The proteins were alkylated by addition of iodoacetamide to a final concentration of 50 mM, followed by 10 min incubation at RT while shaking in the dark. On-bead digestion was done by adding 0.25 μg trypsin and a 2-h incubation at RT. The supernatant was collected after pelleting the beads by centrifugation, an additional 0.1 μg trypsin was added, and proteins were digested overnight at RT. The digest was desalted and concentrated on C18 StageTips without acidification (14). Peptide labelling was done by dimethyl labelling, and StageTips were stored at 4°C until measurement by liquid chromatography with tandem mass spectrometry (LC-MS/MS) (15).

### Mass spectrometry and data analysis

Peptide samples were eluted from StageTips with elution buffer (80% acetonitrile, 0.1% formic acid in ultrapure H_2_O), and light and medium labelled samples for the forward and reverse reaction were combined. Next, the samples were reduced to 10% of the original volume by vacuum concentration and diluted in 0.1% formic acid to ∼12 μl. A total of 5 μl sample was injected, and peptides were separated using an Easy-nLC 1000 liquid chromatography system (Thermo Fisher) using a 7–32% acetonitrile gradient over 120 min at a flow rate of 250 nl/min. Data-dependent measurements of the peptides were performed on a Q-Exactive mass spectrometer (Thermo Fisher). Protein identification and quantification was done in MaxQuant v1.6.0.1 with standard settings and requantify enabled (16). N-terminal acetylation and methionine oxidation modifications were set as variable modifications. Carbamidomethylation was specified as a fixed cysteine modification. Light (+0) and medium (+4) dimethyl labelling on the N-termini and lysine residues was specified under ‘labels’. The MS/MS spectra were searched against a human Uniprot database downloaded in June 2017. Mass tolerance was set at 4.5 and 20 ppm for precursor ion and fragment ions, respectively. False Discovery Rate (FDR) was set at 0.01 for both the peptide and protein level. Two ratio counts were required for protein quantification. Common contaminants and decoy database hits were removed from the resulting MaxQuant proteinGroups file. Protein groups were required to have at least two assigned peptides, of which at least one was a unique peptide, and only protein groups with a quantified ratio for the forward and reverse reaction were considered for further analysis. Statistical significance thresholds were set at Q3 + 1.5 * IQR and Q1 – 1.5 * IQR, and proteins were required to exceed these thresholds in both the forward and reverse run to be labelled as significant. The mass spectrometry proteomics data have been deposited to the ProteomeXchange Consortium via the PRIDE (24) partner repository with the dataset identifier PXD047930.

### Luciferase reporter assay

HEK293T cells were transfected using polyethylenimine (Sigma-Aldrich, 408 719) in a 96-well plate according to the manufacturer’s protocol. Unless stated otherwise, 2 ng TK-Renilla, 5 ng pGL3 reporter, 2 ng pcDNA3.1-RXRα-FLAG or 20 ng pcDNA3.1-RXRβ-FLAG and 10 ng pcDNA3.1 expressing an FXR or GFP was used. A total of 24 h after transfection, cells were incubated with 0.1% DMSO or 10 μM OCA overnight. Next, cells were lysed and the activity of firefly and renilla luciferases were measured with the Dual-Luciferase Reporter Assay System (Promega, E1960) in a TriStar2 LB942 Multimode Reader (Berthold Technologies, Bad Wildbad, Germany). Protein expression of transfected plasmids was verified on western blot. Results are expressed as relative luciferase units, according to the manufacturer’s protocol. For the analysis of FXR or RXR binding to IR-1 or ER-2 motifs in cells, pcDNA3.1 vectors expressing VP16 fusion proteins RXRα-VP16, RXRβ-VP16, FXR-VP16 or GFP-VP16 were used instead, media was changed 24 h after transfection, and cells were lysed and measured 48 h after transfection.

### Split luciferase assay

HEK293T cells were seeded in a white 96 well plate (Corning, 3917) and transfected with 50 ng pBIT2.1-TK_LgBIT-Interactor1 and 50 ng pBIT1.1-TK_SmBIT-Interactor2 using *Trans*IT-LT1 Transfection reagent (Mirus, MIR 2304). Combinations of N-terminal tagged LgBIT:SmBIT interactor pairs used were FXRα2-MycTwinstrep (wt, L434R or L415R): RXRα-FLAG, FXRα2-MycTwinstrep: RXRβ-FLAG, PPARα: RXRα-FLAG or RXRα-FLAG:RXRα-FLAG. The pair LgBIT-PRKAR2A:SmBIT-PRKACA was used as a control vector according to the manufacturers protocol (Promega). A total of 24 h after transfection, the cells were treated in fresh full media as specified in individual figure legends, and before measurement, media was changed to OptiMEM (Gibco, 31 985 062). The Nano-Glo Live Cell Assay System (Promega, N2012) was used according to the manufacturer’s protocol. Luminescence was measured in the TriStar2 LB942 Multimode Reader.

### RNA isolation, reverse transcription followed by quantitative PCR and sequencing

RNA was isolated from HepG2 cells after 8 h treatment (4 μM HX531 + 10 μM OCA) for quantitative PCR (qPCR) analysis, or 16 h treatment (10 μM OCA) for RNA-seq using TRIzol reagent (LifeTechnologies, Naugatuck, CT) according to the manufacturer’s protocol. For reverse transcription followed by qPCR, 500 ng total RNA was reverse transcribed with random hexamers using SCRIPT (Jena biosciences). SsoAdvanced Universal SYBR Green Supermix (Bio-Rad) was used for qPCR on a CFX384 Real-Time system and analysed with the CFX maestro software v2.3 (Bio-Rad). Gene expression was quantified by regression and normalized to PPIA and 18S. Primer pairs were designed using Primer3 or requested from PrimerBank (17) and used at a final concentration of 0.1 μM. Sequences can be found in Supplementary Table S6.

Sequencing libraries were prepared using TruSeq Stranded mRNA Library Prep (Illumina). RNAseq was performed on the NextSeq2000 platform (Illumina). Quality control on the sequence reads from the raw FASTQ files was done with FastQC (v0.11.8). TrimGalore (v0.6.5) was used to trim reads based on quality and adapter presence after which FastQC was again used to check the resulting quality. Reads were aligned to the reference genome GRCh38_snp_tran using HISAT2 (18). Readcounts were generated using the Rsubread FeatureCounts module (v2.0.0) with the Homo_sapiens.GRCh38.102 gtf file as annotation (19). Transcripts per million were calculated per gene using the number of reads count mapped to it over the exon length × 1 million, divided by the total number of mapped reads. Differentially expressed (DE) genes were determined with DESeq2 package (version 1.36.0) (20). Shrinkage of effect size was performed on DEseq2 results using the *apeglm* method through the function lfcShrink. Rsubread, DEseq2 and lfcShrink are Bioconductor packages (Release 3.15) and were executed in RStudio 2022.07.1 Build 554 under R 4.2.0. Enriched known DNA motifs at promoter regions of DE genes and Gene Ontology (GO)-term analysis were performed with HOMER findmotifs (11). Gene-Set enrichment analysis (Preranked) was performed with GSEA v4.2.2 [build 8] with parameters ‘scoring_scheme = weighted, norm = meandiv, include_only_symbols = TRUE, chip = Human_Ensembl_Gene_ID_MSigDB.v2023.1.Hs.chip’ (21,22). RNA-seq data generated in this study are publicly available at the gene expression omnibus (25) under accession number GSE261662.

### Nuclear receptor activity profiling

Ligand-modulated interaction of wt FXRα2 or heterodimerization-deficient mutants with NR-coregulator peptides was assessed using the nuclear receptor activity profiling (NAPing) assay as previously described (23). HEK293T cells were transfected with pCDNA3.1 vectors containing the CDS of FXRα2 wt, L434R or L415R using branched polyethylenimine (Sigma-Aldrich, 408 719) in 15 cm plates. A total of 48 h after transfection, cells were scraped in PBS, washed and snap-frozen and stored at −80°C. Cell lysates of each clone were prepared and FXR was quantified by densitometry of western blot data to enable *in-silico* (equimolar) normalization of NAPing data. A set of immobilized peptides representing coregulator-derived NR binding motifs was incubated with a 25 μl reaction mixture containing 10 μl cell lysate, Tris-buffer saline (TBS: 0.05 M Tris and 0.15 M NaCl, pH 7.6) with 0.05% Tween-20, 0.2% BSA, 50 μM DTT, 10 μM OCA, FXR antibody (Santa Cruz, #sc-25309) and Donkey anti mouse IgG Alexa Fluor 488 (Invitrogen, #A21202). Incubation was performed for 40 min at 20°C, followed by washing to remove unbound receptor/fluorophore and generation of a tiff image of each array. Image processing and quantification of FXR binding to each peptide on the NAPing platform was performed by in-house analysis software based on R [R Core Team (2023). "R: A Language and Environment for Statistical Computing". R Foundation for Statistical Computing, Vienna, Austria.; https://www.R-project.org]. Raw data derived from NAPing assay can be found in Supplementary Data File SD9.

### Statistical analyses

See the relevant section above for description of ChIP-seq and RNA-seq statistical analysis. All other analyses were conducted using Graphpad prism 10.1. t-test with FDR correction were used to compare between groups in NAPing assay. Fisher’s exact test (FET), two-tailed one-way or two-way Analysis of Variance (ANOVA) followed by Tukey multiple comparison correction were used to compare multiple groups. Tests used are noted in the respective figure legends.

## Results

### FXRα2 binds ER-2 motifs independently of RXR

**Figure 1.**
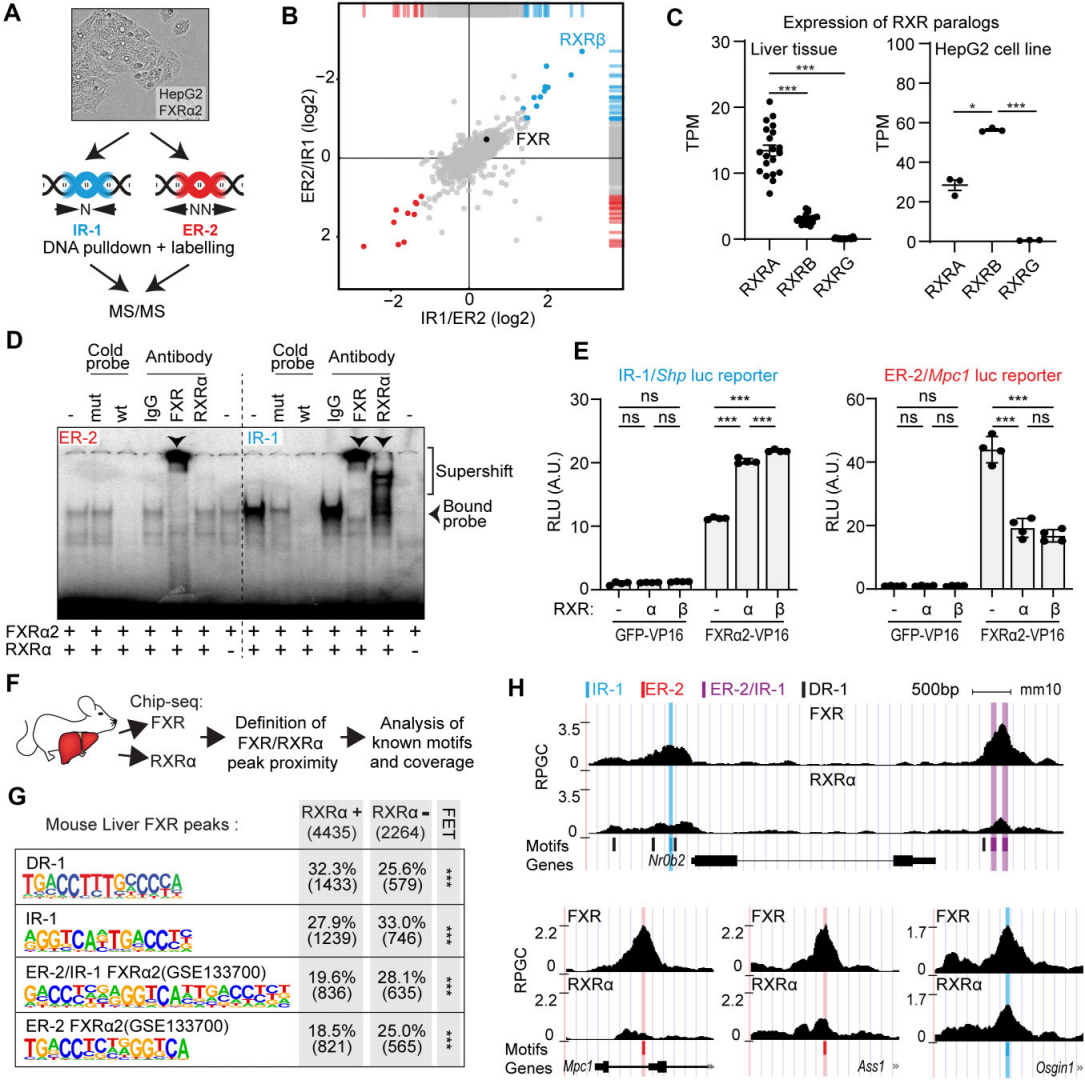
FXRα2 binds ER-2 motifs independently of RXR. (**A**) Workflow for DNA pulldown followed by MS/MS. (**B**) Log2 abundance ratio of proteins enriched in the IR-1 or ER-2 DNA probe pulldowns. (**C**) Expression of RXR paralogs in human liver (GSE77509, *N* = 20) and HepG2 cells (GSE133659, *N* = 3) by RNA-seq. Individual values, average and standard error of the mean are depicted. One-way ANOVA/Tuckey. (**D**) EMSA and super shift assay for human FXRα2 and RXRα on IR-1 and ER-2 probes using *in vitro* translated protein. (**E**) FXRα2-VP16 luciferase reporter assay in HEK293T in the presence of RXRα/β. *N* = 4, two-way ANOVA/Tuckey. Error bars represent standard deviation. (**F**) Schematic workflow of the FXR/RXRα ChIP-seq experiment in mouse liver. (**G**) Enriched DNA motifs in FXR peaks, separated by RXRα co-occupancy. Number of peaks in each category is annotated in parenthesis, FET was used to estimate significance. (**H**) FXR and RXRα occupancy at IR-1, ER-2 or overlapping IR-1/ER-2-regulated loci. Coordinates are found in Supplementary Table S4. *P < 0.05 **P < 0.01, ***P < 0.001, ns, not significant.

We investigated whether the motif preference of FXRα2 is mediated by differential interaction partners at the DNA binding sites. To this end, we performed a DNA pulldown in which immobilized DNA probes containing ER-2 or IR-1 motifs were used to identify differentially interacting proteins in nuclear extracts from HepG2 cells overexpressing FXRα2 (Figure 1A). Following incubation and washes, bound proteins were digested with trypsin, followed by dimethyl-labelling for quantitative detection by mass spectrometry (14). To our surprise, we found RXRβ selectively interacting with IR-1 and not ER-2 (Figure 1B and top hits and ratios in Supplementary Table S7). We then examined whether also RXRα, the predominant RXR paralog in the liver (Figure 1C), is differentially involved in FXRα2 binding to ER-2 or IR-1 DNA motifs in electrophoretic mobility shift assays (EMSA) (Figure 1D and Supplementary Figure S1B and C). FXRα2 binds to both motifs in the presence of RXRα, as previously shown (5,27), and cold probe competition was used to confirm the specificity of FXRα2 binding to the DNA probes. To assess whether RXRα is indeed part of the FXR-DNA complex, we performed supershift assays using antibodies against FXR or RXRα. Normal rabbit IgG was used as a negative control. Using the IR-1 probe, both the RXRα and FXR antibody induced a supershift, confirming the presence of the heterodimer. In addition, as shown in previous studies (24), our results confirm that an FXR:RXR heterodimer is necessary for binding to IR-1 motifs, and neither RXR paralog could bind the IR-1 probe in the absence of FXR (Supplementary Figure S1D). For the ER-2 probe, however, we observed a supershift upon incubation with the anti-FXR but not with the anti-RXRα antibody (Figure 1D, and Supplementary Figure S1B and C). Furthermore, in the absence of RXRα, FXRα2 binding was maintained exclusively with the ER-2 probe, showing that RXRα is neither involved nor required in FXRα2 binding to ER-2 motifs. To verify this observation in a cellular environment, we expressed FXRα2 fused to the VP16 TAD (FXRα2-VP16) and performed luciferase reporter assays to assess binding to the reporters in the presence or absence of RXRα or RXRβ (Figure 1E). In this assay, no ligands are used, whereby the luciferase signal corelates to the binding affinity of the FXRα2-VP16 fusion protein to the reporter construct. The reporter constructs used contained natural IR-1 or ER-2 DNA motifs derived from the mouse *Shp* or *Mpc1* promoters respectively (Supplementary Table S2). Indeed, both RXRα and RXRβ enhanced FXRα2 binding to the IR-1 reporter, seen as an increase in luciferase activity, but reduced binding of FXRα2 to the ER-2 reporter (Figure 1E and Supplementary Figure S1E). Using this assay, we also confirmed that RXRα- and RXRβ-VP16 only bind the IR-1 motif in the presence of FXRα2 (Supplementary Figure S1F and G). Altogether, these data demonstrate that RXR is required for FXRα2 binding to IR-1 but reduces FXRα2 binding to ER-2 motifs.

To evaluate whether *in vivo* FXR:RXR co-occupancy is related to DNA motif enrichment, we performed ChIP-seq for FXR and RXRα in mouse liver tissue (Figure 1F and Supplementary Figure S1H). We identified FXR binding sites, separated them into two subsets based on their overlap with RXRα binding sites, and evaluated the prevalence of DNA motifs in each subset. Both subsets had a significant enrichment for canonical FXR (IR-1) motifs. Co-occupancy of RXRα and FXR showed higher enrichment of the RXR (DR-1, direct repeat spaced by one nucleotide) binding motif. In contrast, FXR peaks without co-occupancy of RXRα were more enriched for IR-1/ER-2 overlapping and ER-2 motifs (Figure 1G and Supplementary Data SD4). In agreement, we also observed higher FXR occupancy relative to RXRα at ER-2 and ER-2/IR-1 sites proximal to FXR target genes, which is not observed at IR-1 sites (Figure 1H and coordinates in Supplementary Table S4). Taken together, we show that RXRα, the canonical heterodimerization partner of FXR, promotes FXRα2 binding towards IR-1 motifs, but is not essential at ER-2 motifs *in vivo*.

### RXR inhibits FXRα2-driven activation from ER-2 motifs

**Figure 2.**
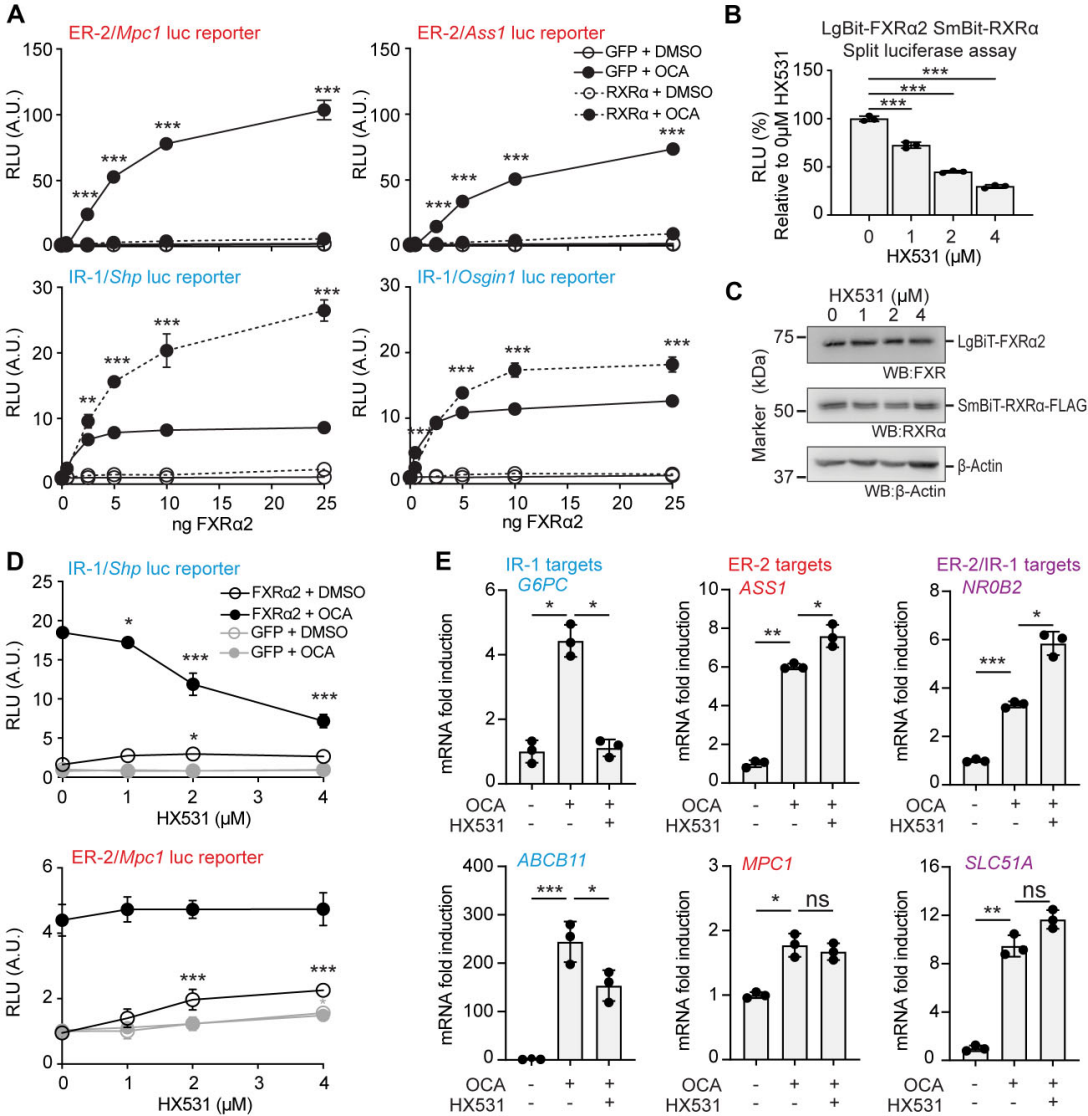
RXR inhibits FXRα2-driven activation from ER-2 motifs. (**A**) Luciferase reporter assays using ER-2- and IR-1-containing reporters in HEK293T, transfected with a titration of FXRα2 in the presence of 10 ng of either GFP or RXRα. *N* = 3, one-way ANOVA/Tukey between matching GFP and RXRα conditions. (**B**) FXRα2:RXRα split luciferase complementation assay in HEK293T cells upon HX531 treatment, *N* = 3. (**C**) Protein expression of FXRα2:RXRα split luciferase components from a representative experiment depicted in panel (B). (**D**) Luciferase reporter assay in HEK293T transfected with 2 ng RXRα and 10 ng of either GFP or FXRα2 upon overnight treatment with DMSO/OCA and HX531. *N* = 3, (B/D) one-way ANOVA/Tukey using 0 μM HX531 as reference. (**E**) Expression of FXR target genes, separated by their proximal FXR RE, in HepG2 cells overexpressing FXRα2 and RXRα upon treatment with OCA and 4 μM HX531 for 8 h. *N* = 3, one way ANOVA/Tukey. *P < 0.05, **P < 0.01, ***P < 0.001. ns, not significant.

To determine the effects of RXRα on FXRα2 transcriptional activity, we performed luciferase reporter assays using natural IR-1 and ER-2 motifs upon treatment with the FXR agonist OCA. Sequences analysed and their respective genomic coordinates are listed in Supplementary Table S2. IR-1 reporters showed enhanced luciferase activity upon OCA treatment in the presence of RXRα. In sharp contrast, FXRα2 activation from ER-2 reporters was strongly inhibited by RXRα (Figure 2A and Supplementary Figure S2A). FXRα4, the other FXR isoform capable of binding to ER-2 motifs (5), presented the same phenotype (Supplementary Figure S2B and C), showing that this mechanism affects both ER-2 binding FXR isoforms. These effects were also observed when RXRβ was used (Supplementary Figure S2B and C), demonstrating redundancy of these RXR paralogs in FXR function at IR-1 and ER-2 sites.

As FXRα2 activation from IR-1 and ER-2 motifs is opposingly regulated by RXR, we hypothesized that interfering with FXRα2:RXRα heterodimerization will have motif-specific effects. HX531 is an RXRα antagonist reported to interfere with RXRα-heterodimer formation with RAR and PPARγ (28,29). We tested the effect of HX531 on FXRα2:RXRα heterodimerization using a split luciferase assay, where luminescence directly correlates with heterodimerization, in live cells (Supplementary Figure S2D). A dose-dependent decrease in luciferase activity was observed (Figure 2B,C), reaching a 70% decrease in heterodimerization at a concentration of 4 μM HX531. This effect was also tested with FXRα2:RXRβ and additional RXR homo- and heterodimers, giving comparable results (Supplementary Figure S2E). Next, we assessed the effect of HX531 in FXRα2 activating from IR-1 and ER-2 luciferase reporter constructs in cells co-transfected with RXRα upon treatment with OCA (Figure 2D). As expected, based on the split luciferase assay, HX531 downregulated FXR activation from the IR-1 reporter in a dose-dependent manner, up to a 50% decrease at 4 μM. For the ER-2 reporter, the basal FXRα2 activity is already reduced due to the presence of overexpressed RXRα, in line with Figure 2A. However, HX531 had no further effect on FXRα2 activity from the ER-2 reporter, providing evidence for the dispensability of RXRα on ER-2 motifs.

To assess whether endogenous FXR targets showed motif-associated RXRα dependence, we performed qPCR on HepG2 cells overexpressing FXRα2 and RXRα. Prior to RNA isolation, the cells were treated with 10 μM OCA or co-treated with 10 μM OCA and 4 μM HX531 for 8 h (Figure 2E and Supplementary Figure S2F). Top FXRα2 targets in HepG2 were extracted from GSE133659 (5), and separated by motif presence at their promoters or proximal regulatory regions (30). Expression of FXR target genes that are regulated through IR-1 motifs was decreased upon HX531 treatment (*ABCB11, G6PC*). However, genes regulated through ER-2 or ER-2/IR-1 overlapping motifs were either not affected by this treatment (*MPC1, SLC51A/OST*α), or even expressed at higher levels in the presence of HX531 (*ASS1, NR0B2/SHP*). In conclusion, interfering with FXRα2:RXRα heterodimerization using the RXRα antagonist HX531 specifically inhibits FXRα2 activation from IR-1 motifs, while FXRα2 activation is maintained or even enhanced at ER-2 and IR-1/ER-2 overlapping motifs.

### L434 in the FXRα2 ligand binding domain is required for RXRα-heterodimerization and activation from IR-1 but not ER-2 DNA motifs

**Figure 3.**
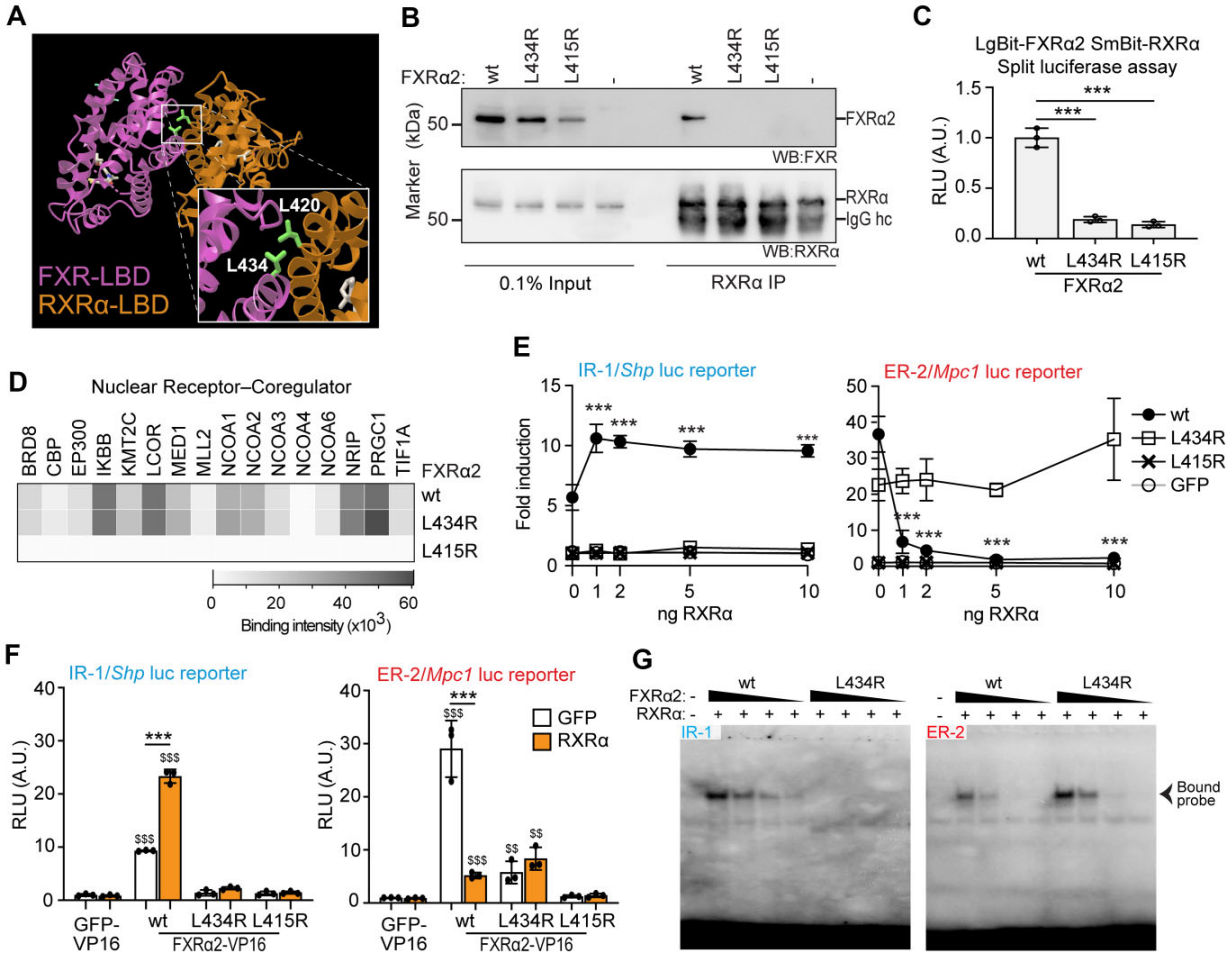
L434 in the FXRα2 LBD is required for RXRα-heterodimerization and activation from IR-1 but not ER-2 DNA motifs. (**A**) FXR:RXRα heterodimerization interface in the LBDs (PDB 5Z12). Most proximal residues according to Zheng *et al.* (31) are highlighted. (**B**) Co-IP of RXRα with either wt FXRα2 or heterodimerization mutants in transfected HEK293T. (**C**) FXRα2:RXRα split luciferase complementation assay in HEK293T. *N* = 3, one-way ANOVA/Tukey. (**D**) Heatmap representing the interaction of wt FXRα2 or mutants with selected NR coregulator peptides (NAPing assay) using lysates from transfected HEK293T in the presence of OCA. (**E**) Luciferase reporter assay with FXRα2 mutants using IR-1 and ER-2 DNA motifs in HEK293T. Values show fold induction between paired OCA/DMSO overnight treatments. *N* = 3, one-way ANOVA/Tukey using matching 0 ng RXRα condition as reference. (**F**) VP16 luciferase reporter assay in HEK293T using FXRα2 mutants in combination with 10 ng of either RXRα or GFP. *N* = 3, one way ANOVA/Tukey. $: comparisons using matching GFP-VP16 condition as reference. (**G**) EMSA assay for a titration of FXRα2^wt^ or FXRα2^L434R^ in the presence of RXRα. $$*P* < 0.01, ***/$$$*P* < 0.001.

Recent structural analyses showed L434 in helix 10/11 in the ligand binding domain (LBD) of FXRα2 is the most exposed and closest residue to RXRα (Figure 3A) (31). We therefore generated an L434R mutant in FXRα2 (FXRα2^L434R^) and assessed whether the FXRα2:RXRα dimerization is affected. For comparison, we also used a FXR with residue L415 mutated to arginine [FXRα2^L415R^ previously annotated as L433R (32)] in helix 9 of the LBD, which is described to be impaired in heterodimerization with RXRα (32). co-IP in HEK293T overexpressing FXRα2^wt^, FXRα2^L434R^ or FXRα2^L415R^ and RXRα, using an antibody against RXRα resulted in the pull-down of FXRα2^wt^, but not of FXRα2^L434R^ or FXRα2^L415R^ (Figure 3B). In line with this, FXRα2^L434R^ and FXRα2^L415R^ showed markedly reduced luciferase activity in comparison to FXRα2^wt^ in split luciferase assays (Figure 3C and Supplementary Figure S3A). In conclusion, both FXRα2^L434R^ and FXRα2^L415R^ are defective in heterodimerizing with RXRα.

We further characterized the RXRα heterodimerization-deficient mutants using a NAPing assay (23). We found that in the presence of OCA, FXRα2^wt^ and FXRα2^L434R^ share the same coactivator binding profile. In strong contrast, FXRα2^L415R^ does not show detectable binding to any coregulator-derived motif (Figure 3D and full results in Supplementary Figure S5), suggestive of an LBD folding defect. Activation of luciferase reporters by FXRα2^wt^ and mutants upon treatment with OCA showed that only FXRα2^wt^ could activate from the IR-1 reporter, and this activation was further enhanced by RXRα. Both FXRα2^wt^ and FXRα2^L434R^ could activate from the ER-2 reporter. However, co-transfection of RXRα repressed FXRα2^wt^ but not FXRα2^L434R^ demonstrating that FXRα2^L434R^ is an RXRα-insensitive mutant that maintains ER-2 transactivation capability. As expected, FXRα2^L415R^ could not activate from either motif in the luciferase reporter constructs (Figure 3E and Supplementary Figure S3B).

To evaluate the DNA binding specificity of the mutants, we used FXRα2-VP16 fusions in the presence or absence of RXRα in luciferase reporter assays. FXRα2^wt^ showed binding to the IR-1 motif, which is enhanced by RXRα, and to the ER-2 motif, which is repressed by RXRα (Figure 3F and Supplementary Figure S3C). Interestingly, FXRα2^L434R^ was only capable of binding to the ER-2 reporter, albeit less than FXRα2^wt^, and the binding was not affected by RXRα. FXRα2^L415R^ did not bind to either IR-1 or ER-2 reporter, suggesting a DNA binding impairment in addition to a co-activator binding deficiency under our testing conditions (Figure 3D). We further characterized FXRα2^L434R^ binding to DNA in EMSA using a titration of FXRα2 in the presence of RXRα (Figure 3G). We observed no binding of FXRα2^L434R^ to the IR-1 probe, in contrast to FXRα2^wt^. Using the ER-2 probe however, FXRα2^L434R^ binding was comparable to FXRα2^wt^ binding. In conclusion, we have generated an FXRα2 mutant in the LBD (FXRα2^L434R^) that selectively binds and activates transcription from ER-2 motifs and is insensitive to the inhibitory effect of RXRα at these motifs.

### RXRα-independent effects of FXRα2 occur at ER-2-regulated genes transcriptome-wide

**Figure 4.**
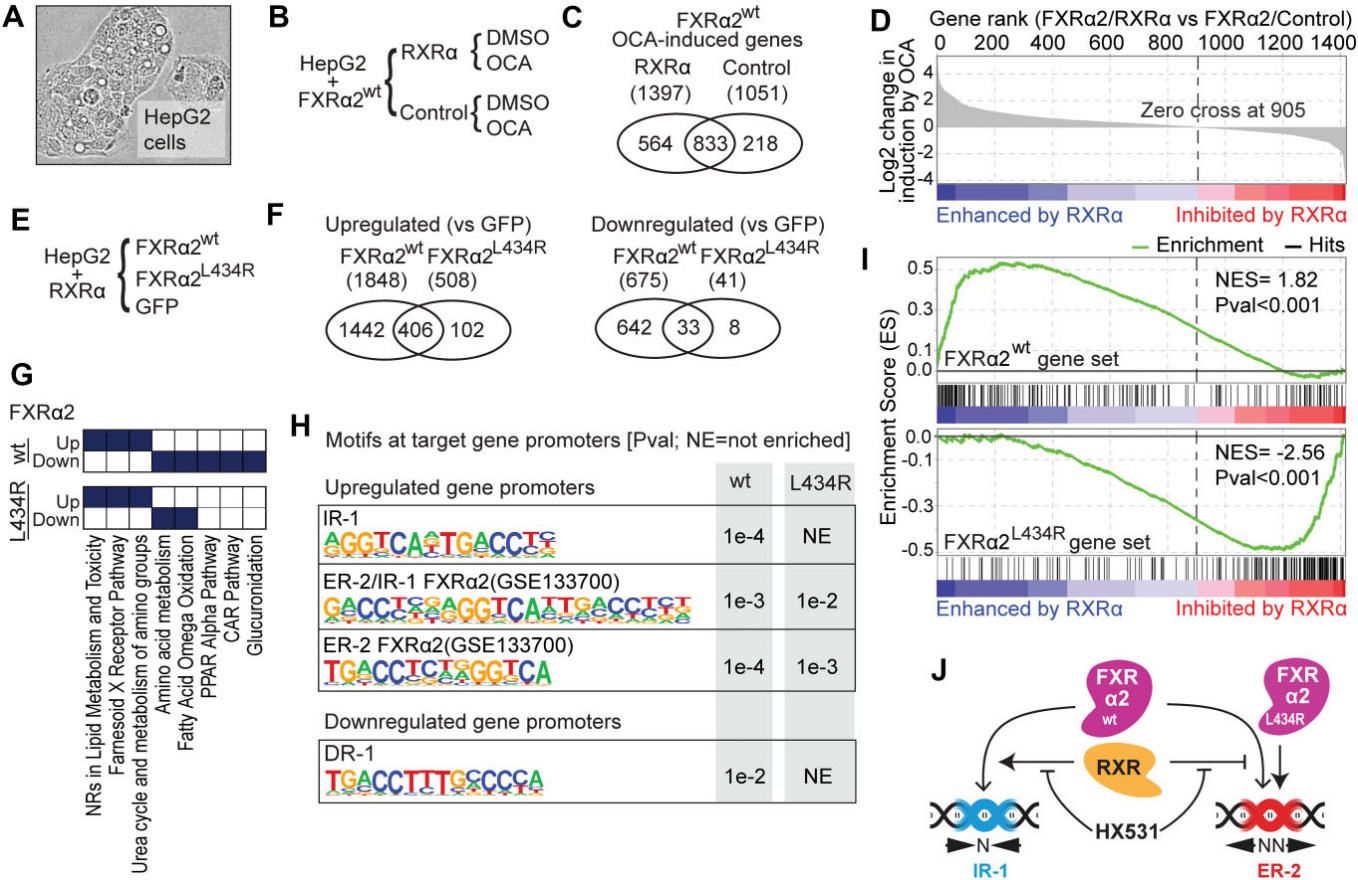
RXRα-independent effects of FXRα2 occur at ER-2-regulated genes transcriptome-wide. (**A**) Phase contrast image of HepG2 cells. (**B**) Overview of generated cell lines and workflow to analyze RXRα dependence of FXRα2 target genes. (**C**) Venn diagram representing the overlap of OCA-induced target genes with or without RXRα overexpression. Total number of genes upregulated per cell line is noted in parenthesis. (**D**) FXRα2 target genes, ranked by change in fold-induction upon RXRα overexpression. (**E**) Overview of generated cell lines to identify FXRα2^L434R^ target genes. (**F**) Venn diagrams representing DE genes between OCA-treated cells expressing FXRα2^wt^ or FXRα2^L434R^, in comparison to GFP. Total number of genes upregulated or downregulated per cell line are noted in parenthesis. (**G**) GO term analysis of upregulated and downregulated genes from panel (F). Terms with *P* < 0.01 are coloured in navy. (**H**) HOMER known-motif enrichment in FXRα2^wt^ or FXRα2^L434R^ target gene promoters. (**I**) GSEA Preranked on FXRα2 target genes, ranked by change in fold induction upon RXRα overexpression shown in panel (D), against gene sets containing the top 150 upregulated FXRα2^L434R^ or FXRα2^wt^ target genes. (**J**) Proposed model for RXR as a pivotal factor in DNA motif selectivity of FXRα2, enhancing IR-1 while inhibiting ER-2 responses.

To evaluate the impact of RXRα on FXRα2 activated target genes transcriptome-wide, we generated HepG2 cells overexpressing FXRα2 and either RXRα or a control vector (Figure 4A and B, and Supplementary Figure S4A). The cells were treated with 0.1% DMSO (vehicle) or OCA overnight and RNA sequencing was performed. We started by comparing the number of genes induced by FXRα2 upon OCA treatment, in the presence or absence of RXRα overexpression (Figure 4C). While most upregulated genes (833) were common to both RXRα and control cells, 564 and 218 genes were specifically induced in the presence or absence of RXRα overexpression, respectively. We proceeded to quantitatively evaluate the difference in fold-induction upon OCA stimulation resulting from RXRα overexpression per FXRα2 regulated gene (Figure 4D). A ranked gene list was generated based on the log2 change in induction by OCA, where positive numbers reflect increased induction, and negative numbers reflect decreased induction in RXRα overexpressing cells (Supplementary Data SD5). For this analysis, only genes successfully quantified in all samples by RNA-seq mapping to identifiers present in the Human Ensembl Gene_ID MSigDB.v2023.1 chip in the GSEA software were considered. We found that most genes were enhanced by RXRα overexpression, however, around one quarter were inhibited to 0.5-fold or less. These results show a role for RXRα in FXRα2 target gene specificity transcriptome-wide, where upregulated FXRα2 target genes can be segregated into two groups: enhanced or inhibited by RXRα.

Considering the ER-2 motif specificity and RXRα independence of FXRα2^L434R^, we proceeded to characterize its transcriptomic response to OCA treatment, in comparison to FXRα2^wt^. For this purpose, we used HepG2 cells overexpressing RXRα, and either FXRα2^wt^, FXRα2^L434R^ or GFP (Figure 4E). These cells were treated with OCA overnight and RNA sequencing was performed (Supplementary Figure S4B). We then assessed differences between OCA-treated FXRα2^wt^ and FXRα2^L434R^ compared to the GFP-expressing cell line. FXRα2^wt^ activation resulted in 3.6-fold more upregulated genes, and 16.5-fold more downregulated genes compared to FXRα2^L434R^ (Figure 4F). However, most FXRα2^L434R^ driven changes (406 out of 508) were shared with FXRα2^wt^, in line with FXRα2^wt^ being able to activate from ER-2 sites as well. We next performed GO term analysis on genes activated or repressed by FXRα2^wt^ or FXRα2^L434R^ as shown in Figure 4F, against the Molecular Signatures Database (Figure 4G and Supplementary Data SD6). Interestingly, terms depicting known FXR functions (e.g. lipid metabolism and ammonia detoxification) were enriched in both FXRα2^wt^ and FXRα2^L434R^ cell lines (33). On the other hand, pathways related to other RXR-heterodimerization partners CAR and PPARA were downregulated exclusively by FXRα2^wt^, raising the possibility that FXR may be competing with CAR and PPAR to heterodimerize with RXR upon treatment with OCA.

To confirm the link between DNA motif selectivity and RXR-independent FXRα2 functions, we tested for motif enrichment at the promoters of FXRα2^wt^ or FXRα2^L434R^ target genes (Figure 4H and Supplementary Data SD7) (33). The ER-2 and IR-1 motifs were the highest enriched amongst promoter regions of FXRα2^wt^ upregulated genes, followed by the ER-2/IR-1 overlapping motif. The DR-1 motif was the only motif enriched in FXRα2^wt^ downregulated genes, which is the canonical binding site of numerous RXR homo- and heterodimers, hinting at competition with other NRs for RXR (34). On the other hand, promoter regions of FXRα2^L434R^ upregulated genes were enriched for the ER-2 and ER-2/IR-1, but not for the IR-1 motif, confirming the motif preference for ER-2 in the absence of FXR:RXR heterodimerization genome-wide.

Next, we evaluated the relationship between RXRα-dependency of FXRα2^wt^ target genes and FXRα2^L434R^ targets. We performed GSEAPreranked (21,22) against the list of FXRα2 targets ranked on the changes in induction by OCA upon RXRα overexpression (Figure 4D). To compare, we generated gene sets derived from the 150 most induced genes in the FXRα2^L434R^ or FXRα2^wt^ overexpressing HepG2-RXRα cell lines compared to the GFP-expressing cell line (GSE261662). We observed that genes enhanced by RXRα were enriched for the FXRα2^wt^ gene set, while genes inhibited by RXRα were enriched for the FXRα2^L434R^ gene set (Figure 4I). This shows that FXRα2:RXRα heterodimerization acts as a molecular switch in FXR-driven transcription, promoting activation at IR-1 sites while preventing or inhibiting activation from ER-2 sites. In summary, we show that while RXRα enhances a subset of FXRα2 target genes, it also inhibits another subset of genes. These inhibited genes represent around 30% of FXRα2 targets, are enriched for ER-2 motifs, are activated by FXRα2^L434R^, and are involved in lipid and amino acid metabolism. All in all, our results demonstrate that FXRα2 activating transcription from ER-2 sites is inhibited by RXRα heterodimerization but specifically maintained by the RXR-independent FXRα2^L434R^ at the transcriptomic level (Figure 4J).

## Discussion

We characterized RXR as a major regulator in the differential binding of FXRα2 to the canonical IR-1 and non-canonical ER-2 DNA motifs in liver cells. We discovered that RXR is not involved in FXRα2 binding to ER-2 motifs. Moreover, FXRα2:RXR heterodimerization even inhibits FXRα2 binding to the non-canonical ER-2 motifs.

Recently, Jiang *et al.* showed, using recombinant FXR and RXRα DBDs in an EMSA setting, that the FXR:RXR heterodimer preferably binds to IR-1 or IR-0, while FXR alone binds to IR-0, DR-1 and ER-2 motifs (35). Based on the electrophoretic mobility shift of the FXRα2:IR1 and the FXRα2:ER2 showing complexes comparable in migration distance, we speculate that ER-2 may be bound by an FXRα2 homodimer, akin to the other NRs bound to DNA (36). However, further studies are needed to elucidate the precise full length FXRα2 complex at the different motifs. Our results suggest that the FXRα2:RXR heterodimer binding to IR-1 is favoured over FXRα2 binding to ER-2 motifs (Supplementary Figure S1I). Therefore, in the presence of available RXR, the FXRα2 pool suitable for ER-2 binding would be reduced, leading to a reduction in the activation of these genes (Figures 2A and 4D). Interestingly, while FXRα2 binding to ER-2 motifs is reduced by RXRα co-expression, we and others have found that RXRα induces a higher molecular weight complex at ER-2/IR-1 overlapping motifs, specific for FXRα2 (Supplementary Figure S1J and K) (27). Moreover, FXRα2 retained most of its transactivation capacity in the presence of RXRα using a luciferase reporter containing an ER-2/IR-1 overlapping motif (Supplementary Figure S4C). This suggests that the ER-2/IR-1 overlapping motif is bound by FXRα2 and activated as either ER-2 or IR-1, said otherwise, activated in the presence or absence of RXR without its inhibiting effects. However, the effects of the modulation of FXR:RXR heterodimerization in DNA binding selectivity *in vivo* remains to be elucidated.

In a physiological context, FXR isoform expression in the mouse liver was shown to be regulated through bioenergetic cues, and FXRα2 expression was induced by exercise or fasting (37). Yang *et al.* showed that RXRα expression in the mouse liver follows a rhythmic expression pattern according to feeding times with the lowest RXRα expression during fasting (38). The ER-2-regulated gene program would drive improved lipid clearance, increase ammonia production, and block lipogenesis (33). As activation of such a gene program is likely to be beneficial to combat metabolic diseases, we tentatively propose that this window of low RXR and high FXRα2 expression during fasting may be exploited to enhance gene activation through FXRα2 binding ER-2 DNA binding motifs. On top of that, increased RXR-independent FXRα2 actions may allow for reduced competition with other NRs, such as PPARs, for RXR. Kemper *et al.* showed that constitutive FXR acetylation upon obesity results in reduced FXR:RXRα heterodimerization, DNA binding and transactivation function (39). Whether this reduction in DNA binding and transactivation capacity also affects ER-2 target genes remains unknown.

In a previous study, we determined that 89% of all FXR binding sites in liver organoids were bound exclusively by FXRα2 or FXRα4, and not by FXRα1 or FXRα3, and were enriched for the ER-2 motif (5). A recent report by Zummo *et al.* (40) showed by single-cell RNA-seq that, in the mouse liver, hepatocytes express mostly *Fxrα3* and *Fxrα4*. Here, we show that selective activation from the DNA motifs by FXRα2 and FXRα4 is influenced by RXR in the same way (Supplementary Figure S2B and C), demonstrating that this FXR isoform-specific mechanism should also be of relevance in mouse hepatocytes.

An interesting point is that inhibiting FXRα2:RXRα heterodimerization, via either a mutation in the LBD or ligands, have a strong impact on DNA motif selectivity of FXRα2. This supports the notion of full-length allosterism, modelled for other type II NRs including PPARG and LXRB (41), but so far not functionally shown for full length NRs. This also raises the possibility that small molecule allosteric modulators of FXR (42) may be designed to hinder heterodimerization with RXR, to boost its ER-2 response. Our findings regarding the effect of the L434R mutation in FXRα2 may be relevant for the transcriptional response of other type II NR. Zhang *et al.* (31) and Weikum *et al.* (43) systematically analysed the structure of published RXRα heterodimers showing that helix 10/11 is structurally highly conserved. We further analysed the primary sequences of RXR heterodimerization partners using the multi-aligner Clustal Omega (44). Interestingly, FXRα2_L434 is conserved in several type II NRs, including PPARG, PPARA, PPARD, LXRA, LXRB, RARA, RXRA, RXRB, THRA, THRB and CAR (Supplementary Data SD8). The role of this residue in the dimerization with RXR may also be conserved among other type II NRs, which would compete with FXRα2^wt^, but not FXRα2^L434R^, for heterodimerization with RXR. This would be supported by the higher number of downregulated genes possessing a DR-1 motif at their promoter region in FXRα2^wt^, in contrast to FXRα2^L434R^ (Figure 4F). From the NR we analysed, RARA has also been shown to function as a homo- and heterodimer with RXR (45). However, to our knowledge, the RAR:RAR, RXR:RXR, and RAR:RXR dimers bind to the same array of DR REs (45–47). The mechanism we describe is FXR isoform-specific and relies on alternative selective use of REs by FXRα2/4, where binding to ER-2 motifs is enhanced in the absence of RXRα. It is plausible that more type II NRs may also be capable of RXRα-independent DNA binding at alternative REs.

In conclusion, we discovered that RXR is not required for FXRα2 activation from the non-canonical ER-2 motifs, thereby refuting the current dogma of FXR being an obligate heterodimer partner of RXR. Modulation of FXR isoform expression or heterodimerization capacities, regulated by circadian and bioenergetic cues, represent promising features to exploit in the optimization of FXR agonism in the treatment of metabolic diseases.

## Supplementary Material

gkae1214_Supplemental_Files

## Data Availability

The mass spectrometry proteomics data have been deposited to the ProteomeXchange Consortium via the PRIDE (24) partner repository with the dataset identifier PXD047930. ChIP-seq and RNA-seq data generated in this study are publicly available at the gene expression omnibus (25) under accession numbers GSE73624 and GSE261662. Previously published RNA-seq reads from HepG2 or human liver biopsies were accessed from GSE133659 (5) and GSE77509 (26) respectively, and re-analysed as described in the RNA-seq analysis section. Raw data derived from NAPing assay can be found in Supplementary Data File SD9.
