# Correction to ‘The farnesoid X receptor activates transcription independently of RXR at non-canonical response elements’

**DOI:** 10.1093/nar/gkag910

**Published:** 2026-09-15

This is a correction to: Suzanne W van der Veen, Jelmer J Dijkstra, Ellen C L Willemsen, René Houtman, Alexandra Milona, Nikolas Marchet, Maureen Spit, Danielle Hollman, Fried J T Zwartkruis, Michiel Vermeulen, Jose M Ramos Pittol, Saskia W C van Mil, The farnesoid X receptor activates transcription independently of RXR at non-canonical response elements, *Nucleic Acids Research*, Volume 53, Issue 4, 28 February 2025, gkae1214, https://doi.org/10.1093/nar/gkae1214

In the originally published version of this manuscript, a statement was omitted from the ‘Funding’ section. The omitted statement reads: “The M.V. lab is part of the Oncode Institute, which is partly funded by the Dutch Cancer Society (KWF)”.

Additionally, in Figure S1E, the clipping mask of a western blot image was shifted, which affected the position of the displayed membrane. The corrected Figure S1E is included below:

**Figure:**
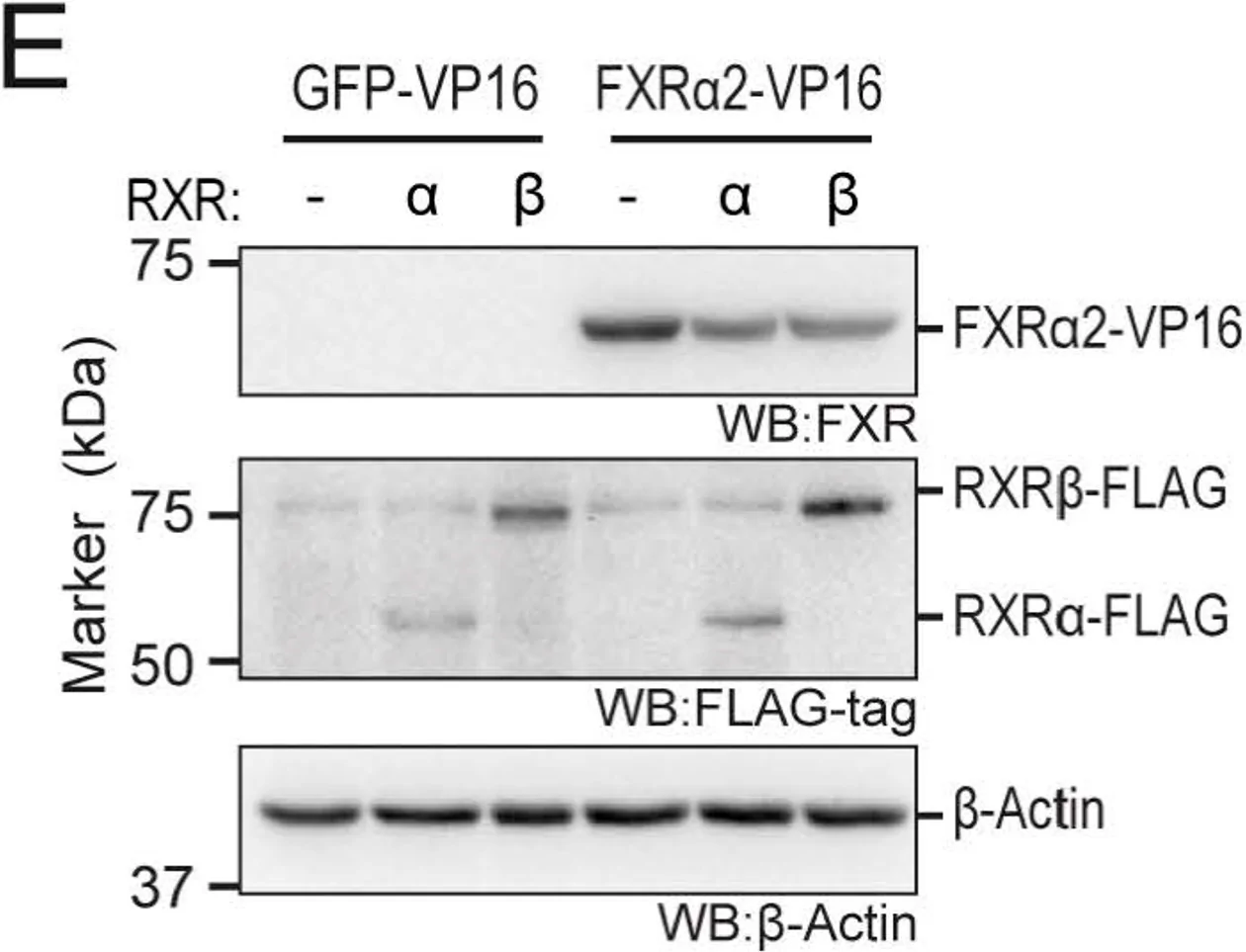


These details have been corrected only in this correction notice to preserve the version of record.

