# Supplementary material for: The farnesoid X receptor activates transcription independently of RXR at non-canonical response elements
Source: Nucleic Acids Res. 2024 Dec 9;53(4):gkae1214. doi: 10.1093/nar/gkae1214 (PMC11879013; doi:10.1093/nar/gkae1214)
Supplement: gkae1214_Supplemental_Files [file gkae1214_supplemental_files.zip › SD1-Supplementary figures and tables.pdf]

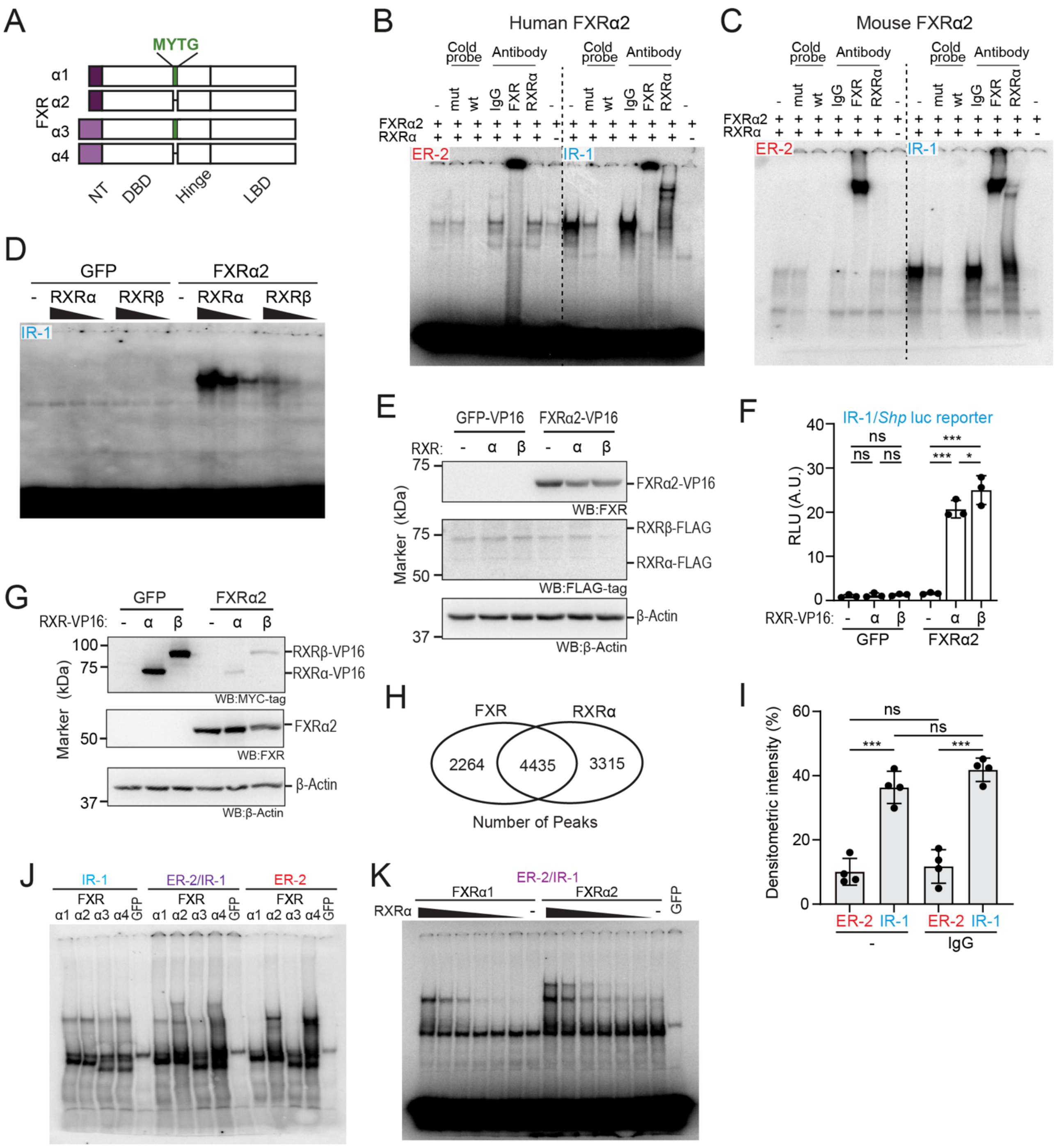

Figure S1. A) FXR isoforms. Annotated are the amino terminal domain (NT), the DNA binding domain (DBD), the MYTG insertion, the Hinge domain, and the ligand binding domain (LBD). Not on scale. B/C) EMSA and super shift assay for FXR and RXRα using human (B) or mouse (C) FXRα2 on ER-2 or IR-1 probes. D) EMSA assay using the IR-1 probe and a titration of RXR paralogs in the presence or absence of FXRα2. E) Protein expression of FXRα2-VP16 and RXR paralogs from a representative luciferase reporter assay shown in figure 1E. F) Luciferase reporter assay on IR-1 reporter in HEK293T of RXR paralogs fused to VP16 in the presence or absence of FXRα2. N=4, Two-way ANOVA/Tuckey. G) Protein expression of RXR paralogs-VP16 fusions (containing myc-tag, as described in materials and methods) and FXRα2 from a representative luciferase reporter assay of panel S1D. H) Overlap of FXR and RXRα peaks in mouse liver. I) Densitometric quantification of FXR binding to ER-2 or IR-1 probes in the presence or absence of IgG. N=4, Two-way ANOVA/Tuckey. J) EMSA assay for human FXR isoforms in the ER-2/IR-1 motif series

sequences. K) Effect of a titration of RXRα in FXR binding to ER-2/IR-1 probe by EMSA. * p<0.05, *** p<0.001. Error bars represent SD.

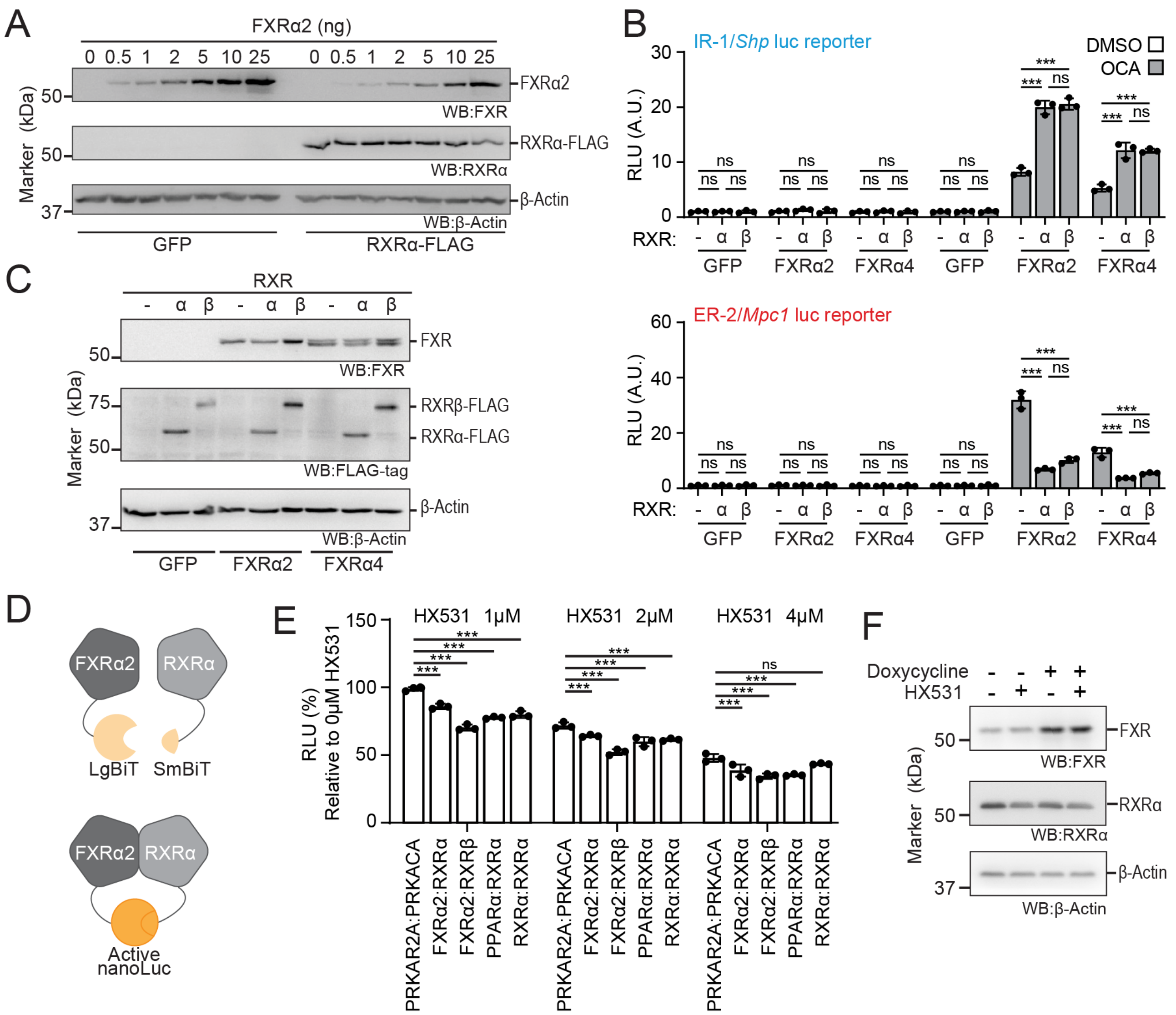

Figure S2. A) Western blot control for FXR and RXRα protein expression of a representative luciferase reporter assay shown in figure 2A. B) Effect of RXR paralogs in FXRα2 and FXRα4 activation in luciferase reporter assays in HEK293T upon treatment with OCA. C) Protein expression of FXR isoforms and RXR paralogs by western blot for luciferase reporter assay depicted in panel S2B. D) Schematic representation of split-luciferase complementation assay. E) Split luciferase complementation assay of RXR dimerization partners in HEK293T cells upon 4 hours HX531 treatment. N=3, two way ANOVA/Tukey using PRKAR2A:PRKACA as a reference. Interacting pairs are annotated according to their tag in the order LgBIT:SmBIT. F) Protein expression of FXR and RXRα after 3 days doxycycline induction and 8 hours HX531 treatment in HepG2 cells, related to figure 2E. ** p<0.01, *** p<0.001, ns=not significant.

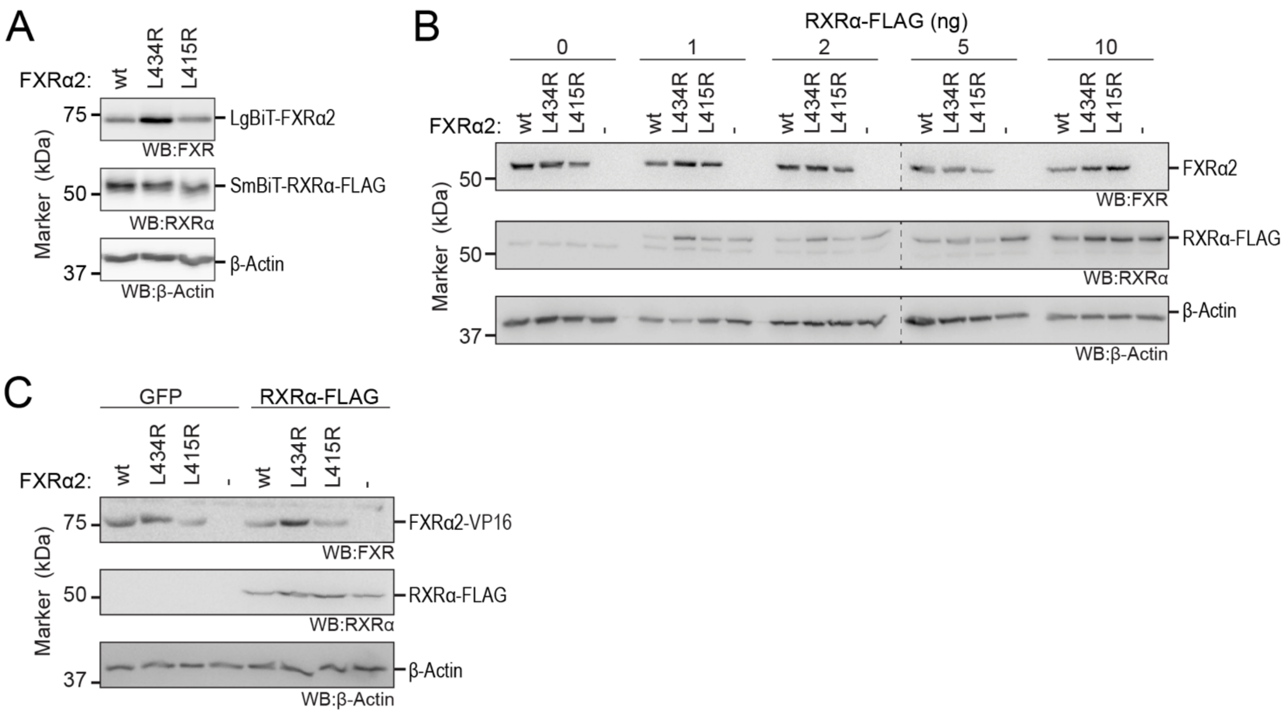

Figure S3. A) Protein expression of LgBiT- fused FXRα2 wt and heterodimerization deficient mutants, and SmBit-RXRα expression from a representative split-luciferase assay depicted in figure 3C. B) Representative western blot control for FXRα2 wt and heterodimerization-deficient mutants, and RXRα for the luciferase reporter assay depicted in figure 3E. C) Representative Western blot control for FXRα2 wt and heterodimerization-deficient mutants fused to VP16, and RXRα for the luciferase reporter assay from figure 3F.

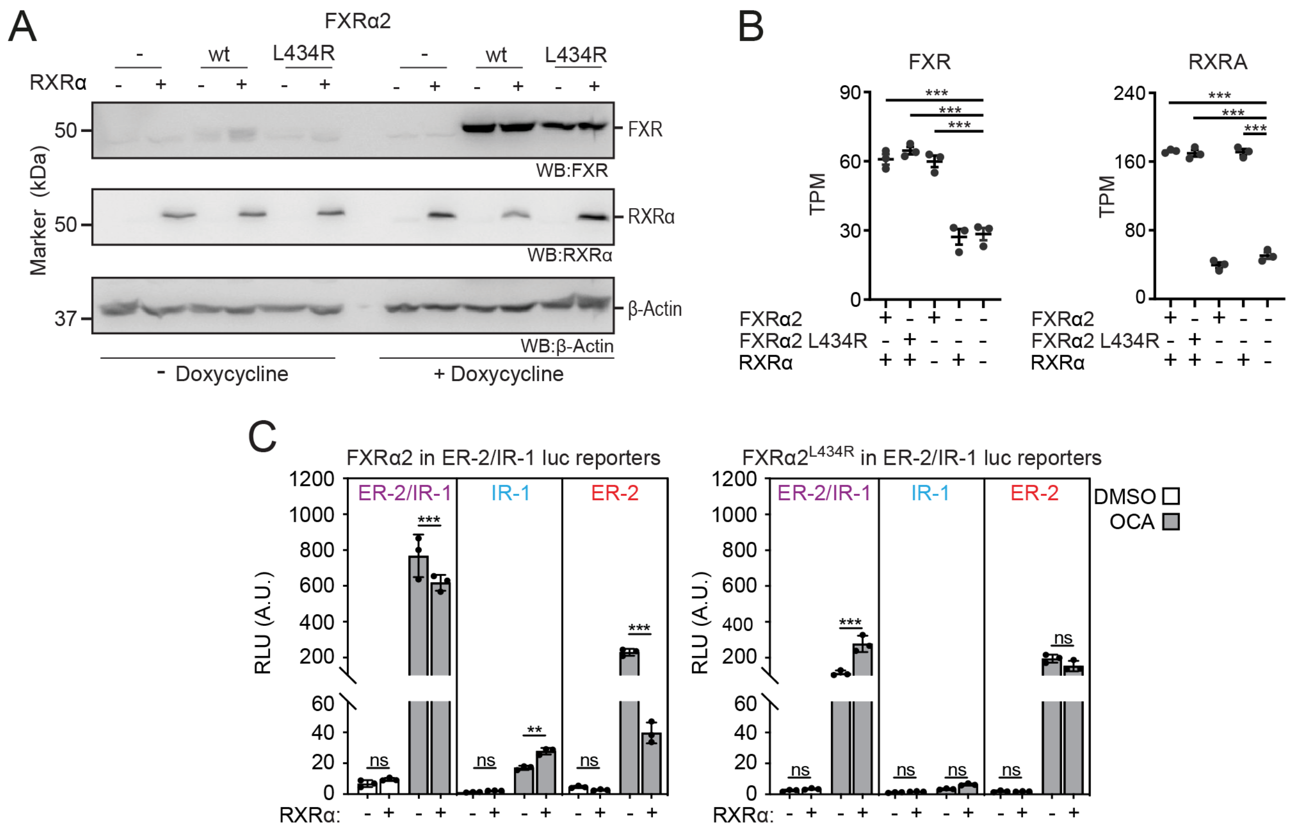

Figure S4. A) Western blot control for FXR and RXRα expression after doxycycline induction in HepG2 cell lines depicted in figure 4B and 4E. B) Expression of FXR and RXRα after doxycycline induction in HepG2 cell lines depicted in figure 4B and 4E by RNA-seq. N=3, One way ANOVA / Tukey. C) Effect of RXRα in FXRα2 wt or L434R activity at the ER-2/IR-1 motif series sequences upon treatment with OCA by luciferase reporter assay in HEK293T. N=3, two way ANOVA/Tukey. *** $p<0.001$, ns=not significant.

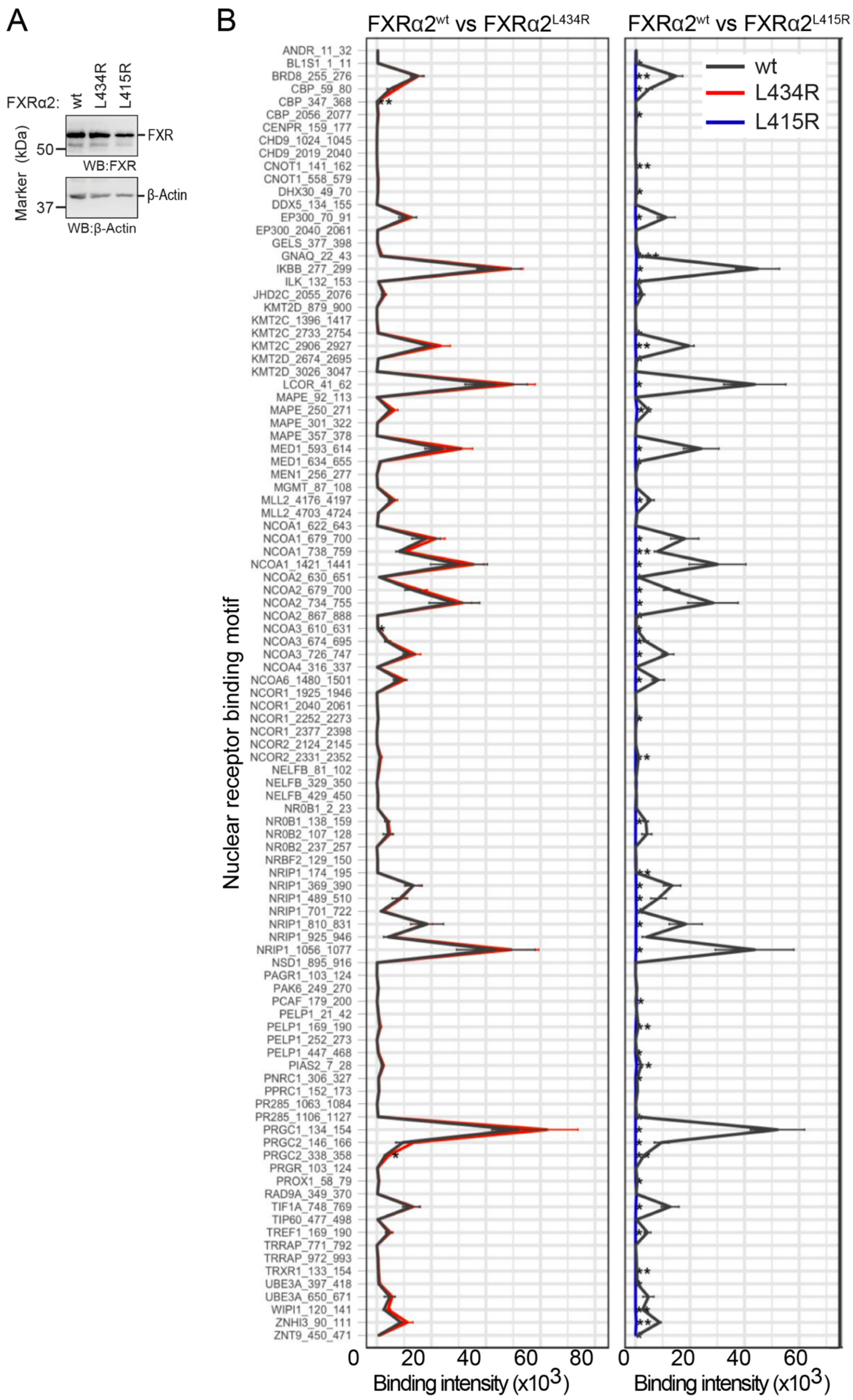

Figure S5. A) Western blot control for FXR expression in transfected HEK293T cells used for the NAPing assay. Only FXRα2 wt, L434R and L415R were used. B) NAPing assay on FXRα2 wt and heterodimerization-deficient mutants expressed in Hek293T cells in the presence of OCA. N=3, t-test, mean binding +/- SEM is plotted. * p<0.05, ** p<0.01.

Table S1. Primers used for mutagenesis of FXRα2

Name	Sequence
FXRa2-L434R(T1301G)_Fw	CAGTCAGGCGACCCCGGAGACAGGCAAAG
FXRa2-L434R(T1301G)_Rv	CTTTGCCTGTCTCCGGGGTCGCCTGACTG
FXRa2-L415R(T1244G)_Fw	GAATCTTACACAACTTTTGTCGCACATCAAGAAGTGGCTCC
FXRa2-L415R(T1244G)_Rv	GGAGCCACTTCTTGATGTGCGACAAAAGTTGTGTAAGATTC

Table S2. Sequences cloned in pGL3 luciferase reporter vectors and their coordinates (mm10)

ID	Chr	Start	End	Sequence	Motif
Ass1	chr2	31464813	31464836	GGTCTGGCCTGGAGGTCATTCT	ER-2
Mpc1	chr17	8284413	8284436	CAAGTGACCGAGCGGACAGCGC	ER-2
Osgin1	chr8	119437022	119437042	ACAAGTTCATTCACCCAGGG	IR-1
Shp_pro	chr4	133553064	133553085	ACTGGGTTAATGACCCTGTT	IR-1
Shp_enh	chr4	133557505	133557529	CAGGGTGACTGACCTGAAGGGTGAG	ER-2/IR-1
	chr2	31464813	31464836	CAGGGacACTGACCTGAAGGGTGAG	ER-2
	chr17	8284413	8284436	CAGGGTGACTGACCTGAAaaGTGAG	IR-1

Table S3. Oligonucleotide probes used for EMSA assays. NR hexamers are shown underlined, inactivating mutations in hexamers are shown in lower case.

Single motif probes	
**Name**	**Sequence**
ER2/mMpc1-wt_Fw	AAGTGACCGAGCGGACAGCG
ER2/mMpc1-wt_Rv	CGCTGTCCGCTCGGTCACTT
ER2/mMpc1-mut_Fw	AAGTGAaaGAGCGGACAGCG
ER2/mMpc1-mut_Rv	CGCTGTCCGCTCttTCACTT
IR-1/mShp-wt_Fw	CCTGGGTTAATGACCCTGTT
IR-1/mShp-wt_Rv	AACAGGGTCATTAACCCAGG
IR-1/mShp-mut_Fw	CCTGGGTTAATGAaaCTGTT
IR-1/mShp-mut_Rv	AACAGttTCATTAACCCAGG
**ER-2/IR-1 motif probes**	
**Name**	**Sequence**
ER-2/IR-1_Fw	GACTGCCCGAGAGGTCAGCGACCTGCC
ER-2/IR-1_Rv	GGCAGGTCGCTGACCTCTCGGGCAGTC
IR-1_Fw	GACTGCaaGAGAGGTCAGCGACCTGCC
IR-1_Rv	GGCAGGTCGCTGACCTCTCttGCAGTC
ER-2_Fw	GACTGCCCGAGAGGTCAGattCCTGCC
ER-2_Rv	GGCAGGaatCTGACCTCTCGGGCAGTC

Table S4. Genomic coordinates for selected FXR-bound regions in figure 1 (mm10)

Locus	Chr	Start	End	Motif
Ass1_Enhancer	chr2	31463866	31465816	ER-2
Mpc1	chr17	8283354	8285311	ER-2
Nr0b2_Enhancer	chr4	133556564	133558583	ER-2/IR-1
Nr0b2_promoter	chr4	133551934	133553953	IR-1
Osgin1	chr8	119431651	119433701	IR-1

Table S5. Probes used for DNA pulldown experiments. NR hexamers are shown underlined.

Name	Sequence
FXR_5AzIR1_Fw	/5AzideN/agtgatttgatGACTGCaaGAGAGGTCAGCGACCTGCCttgcttttgaa
FXR_IR1_Rv	ttcaaaagcaaGGCAGGTCGCTGACCTCTCttGCAGTCatcaaatcact
FXR_5AzER2_Fw	/5AzideN/agtgatttgatGACTGCCCGAGAGGTCAGattCCTGCCttgcttttgaa
FXR_ER2_Rv	ttcaaaagcaaGGCAGGaatCTGACCTCTCGGGCAGTCatcaaatcact

Table S6. Primers used for RT-qPCR

Gene	Forward	Reverse
18S	GATGGGCGGCGGAAAATAG	GCGTGGATTCTGCATAATGGT
ABCB11	TGACTACGACGTTGAGTTACAAG	CTGCGGCAATGACCCAAAA
ASS1	GGGAGTCCCCACTGTCTCT	GTGGCATCAGTTGGCTCAT
G6PC	GGTGTATACTACGTGATGGTCACA	CTGACAGGACTCCAGCAACAA
MPC1	ACTATGTCCGAAGCAAGGATTTC	CGCCCACTGATAATCTCTGGAG
NR0B2	AGCCCAGGCAGAGATCAG	CCAGGAGCATTGGGTCAC
PPIA	CGGGTCCTGGCATCTTGTCC	GCCAAACACCACATGCTTGCC
SLC51A	CTGAAGACCAATTACGGCATC	GAGGGCAAGTTCCACAGG

Table S7. Identified IR-1 and ER-2 interactors from DNA pulldown experiments in figure 1B

Top IR-1 interactors		
Gene.Name	log_forward (IR-1/ER-2)	log_reverse (ER-2/IR-1)
RXRB	2.889746091	-2.713497314
CNBP	2.610865391	-2.114160157
SNAI2	1.981012358	-2.33752103
DNTTIP1	2.019417261	-1.799721654
C14orf43	1.949871056	-1.811320565
CCAR1	1.931569603	-1.706653061
RBM14	1.825337895	-1.557242242
RPS10	1.666393032	-1.542752438
CSTF2	1.73061822	-1.322974428
ZBTB44	1.389401608	-1.259771836
U2AF1	1.500751074	-1.019024187
FIP1L1	1.451646328	-1.024560572
Top ER-2 interactors		
Gene.Name	log_forward (IR-1/ER-2)	log_reverse (ER-2/IR-1)
TEAD1	-2.68315915	2.239825715
TEAD4	-1.801279559	2.19200474
HIC2	-1.655216943	2.131523636
SIX1	-1.913379259	1.626112011
ELF2	-1.393008278	1.427445268
MAZ	-1.558431534	1.396543134
SIX4	-1.854700792	1.314464516
ZNF397	-1.334534459	1.150884571
SIX2	-1.361601738	1.128491379
DCD	-1.209761775	0.969086007
