# Supplementary material for: The farnesoid X receptor activates transcription independently of RXR at non-canonical response elements
Source: Nucleic Acids Res. 2024 Dec 9;53(4):gkae1214. doi: 10.1093/nar/gkae1214 (PMC11879013; doi:10.1093/nar/gkae1214)
Supplement: gkae1214_Supplemental_Files [file gkae1214_supplemental_files.zip › SD3-FXR-RE motifs.rtf]

>AGGTCANTGACCTB	FXR(NR),IR1/Liver-FXR-ChIP-Seq(Chong_et_al.)/Homer	6.873163	-9.761421	0	T:117.0(8.39%),B:2577.2(5.76%),P:1e-4
0.532	0.067	0.360	0.041
0.064	0.001	0.862	0.073
0.025	0.013	0.835	0.127
0.060	0.151	0.167	0.622
0.005	0.859	0.079	0.057
0.907	0.028	0.041	0.024
0.262	0.223	0.260	0.255
0.027	0.066	0.035	0.872
0.054	0.076	0.862	0.008
0.622	0.160	0.152	0.066
0.128	0.836	0.013	0.023
0.053	0.890	0.001	0.056
0.053	0.338	0.072	0.537
0.138	0.347	0.250	0.265

>TGACCTCTRGGTCA	FXRa2(NR)_GSE133700,ER2-motif_15nt(Ramos-Pittol_et_al.)	7.059849	-10.585392	0	T:135.0(9.68%),B:3007.4(6.72%),P:1e-4
0.001	0.124	0.019	0.856
0.110	0.024	0.861	0.005
0.554	0.128	0.204	0.114
0.076	0.887	0.009	0.028
0.033	0.924	0.001	0.042
0.043	0.237	0.119	0.601
0.034	0.670	0.258	0.038
0.266	0.246	0.066	0.421
0.354	0.114	0.414	0.118
0.042	0.001	0.867	0.090
0.038	0.009	0.882	0.071
0.052	0.109	0.252	0.587
0.014	0.865	0.026	0.095
0.919	0.001	0.061	0.019

>GACCYNRRGGTCAHTGACCYYD	FXRa2(NR)_GSE133700,ER2/IR1-overlapping-motif_22nt(Ramos-Pittol_et_al.)	8.169177	-8.894833	0	T:85.0(6.09%),B:1783.7(3.99%),P:1e-3
0.164	0.047	0.744	0.045
0.498	0.219	0.172	0.110
0.119	0.812	0.031	0.038
0.132	0.796	0.001	0.071
0.079	0.397	0.078	0.446
0.245	0.341	0.205	0.209
0.233	0.205	0.367	0.195
0.501	0.062	0.385	0.052
0.046	0.001	0.867	0.086
0.024	0.012	0.900	0.064
0.090	0.148	0.129	0.633
0.007	0.891	0.028	0.074
0.857	0.031	0.083	0.029
0.254	0.229	0.167	0.350
0.131	0.138	0.143	0.588
0.197	0.164	0.527	0.112
0.447	0.292	0.167	0.095
0.176	0.632	0.083	0.109
0.126	0.624	0.057	0.193
0.133	0.350	0.100	0.417
0.130	0.376	0.217	0.277
0.217	0.184	0.257	0.341
