# Supplementary material for: The farnesoid X receptor activates transcription independently of RXR at non-canonical response elements
Source: Nucleic Acids Res. 2024 Dec 9;53(4):gkae1214. doi: 10.1093/nar/gkae1214 (PMC11879013; doi:10.1093/nar/gkae1214)
Supplement: gkae1214_Supplemental_Files [file gkae1214_supplemental_files.zip › SD4-Motifs in liver FXR and RXRA peaks.pdf]

Homer *de novo* Motif Results (FXR/)

Known Motif Enrichment Results
Gene Ontology Enrichment Results
If Homer is having trouble matching a motif to a known motif, try copy/pasting the matrix file into STAMP
More information on motif finding results: HOMER | Description of Results | Tips
Total target sequences = 6697
Total background sequences = 42849
* - possible false positive

Rank	Motif	P-value	log P-pvalue	% of Targets	% of Background	STD(Bg STD)	Best Match/Details	Motif File
1	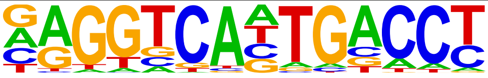	1e-865	-1.994e+03	28.64%	4.98%	45.0bp (63.1bp)	FXR(NR),IR1/Liver-FXR-ChIP-Seq(Chong_et_al.)/Homer(0.995) More Information | Similar Motifs Found	motif file (matrix)
2	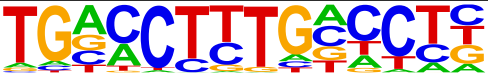	1e-489	-1.128e+03	42.99%	17.95%	50.7bp (60.7bp)	PPARa(NR),DR1/Liver-Ppara-ChIP-Seq(GSE47954)/Homer(0.958) More Information | Similar Motifs Found	motif file (matrix)
3	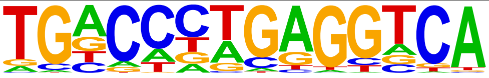	1e-229	-5.274e+02	12.38%	3.27%	47.3bp (63.0bp)	THRb(NR)/Liver-NR1A2-ChIP-Seq(GSE52613)/Homer(0.756) More Information | Similar Motifs Found	motif file (matrix)
4	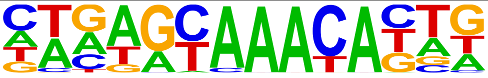	1e-164	-3.790e+02	13.07%	4.58%	52.0bp (61.9bp)	FOXM1(Forkhead)/MCF7-FOXM1-ChIP-Seq(GSE72977)/Homer(0.918) More Information | Similar Motifs Found	motif file (matrix)
5	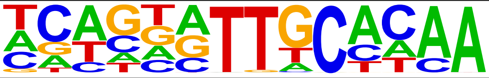	1e-150	-3.475e+02	15.71%	6.49%	52.5bp (61.9bp)	CEBPA/MA0102.3/Jaspar(0.910) More Information | Similar Motifs Found	motif file (matrix)
6	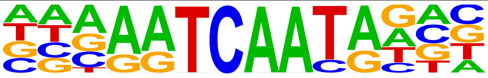	1e-128	-2.967e+02	5.64%	1.21%	51.8bp (59.2bp)	Cux2(Homeobox)/Liver-Cux2-ChIP-Seq(GSE35985)/Homer(0.917) More Information | Similar Motifs Found	motif file (matrix)
7	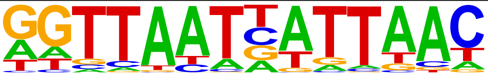	1e-86	-1.989e+02	5.33%	1.53%	54.7bp (58.7bp)	HNF1A/MA0046.2/Jaspar(0.963) More Information | Similar Motifs Found	motif file (matrix)
8	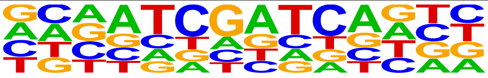	1e-40	-9.373e+01	9.81%	5.64%	52.1bp (59.3bp)	CUX1/MA0754.1/Jaspar(0.843) More Information | Similar Motifs Found	motif file (matrix)
9	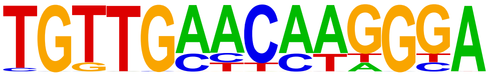	1e-30	-6.999e+01	0.27%	0.00%	40.9bp (0.0bp)	PB0061.1_Sox11_1/Jaspar(0.603) More Information | Similar Motifs Found	motif file (matrix)
10	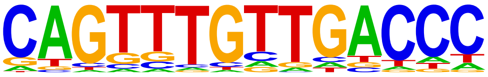	1e-27	-6.314e+01	0.75%	0.09%	40.4bp (75.5bp)	NR4A2::RXRA/MA1147.1/Jaspar(0.677) More Information | Similar Motifs Found	motif file (matrix)
11	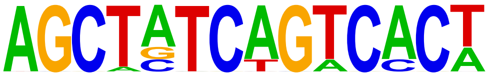	1e-19	-4.566e+01	0.22%	0.01%	46.2bp (14.6bp)	PBX1(Homeobox)/MCF7-PBX1-ChIP-Seq(GSE28007)/Homer(0.679) More Information | Similar Motifs Found	motif file (matrix)
12	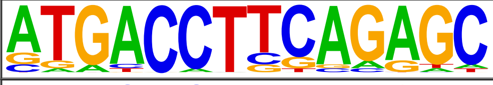	1e-19	-4.456e+01	0.30%	0.02%	44.6bp (37.8bp)	Esrrg/MA0643.1/Jaspar(0.778) More Information | Similar Motifs Found	motif file (matrix)
13	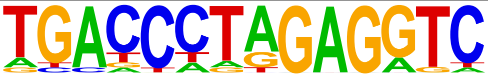	1e-18	-4.193e+01	0.31%	0.02%	46.1bp (27.8bp)	COUP-TFII(NR)/Artia-Nr2f2-ChIP-Seq(GSE46497)/Homer(0.615) More Information | Similar Motifs Found	motif file (matrix)
14	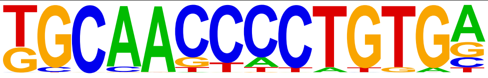	1e-18	-4.161e+01	0.27%	0.01%	51.1bp (47.0bp)	Ddit3::Cebpa/MA0019.1/Jaspar(0.632) More Information | Similar Motifs Found	motif file (matrix)
15	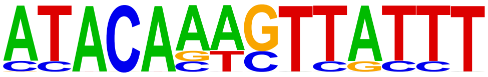	1e-17	-3.973e+01	0.22%	0.01%	51.6bp (28.9bp)	DMRT1(DM)/Testis-DMRT1-ChIP-Seq(GSE64892)/Homer(0.603) More Information | Similar Motifs Found	motif file (matrix)
16	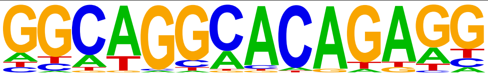	1e-15	-3.626e+01	0.21%	0.01%	37.8bp (24.0bp)	AR-halfsite(NR)/LNCaP-AR-ChIP-Seq(GSE27824)/Homer(0.766) More Information | Similar Motifs Found	motif file (matrix)
17	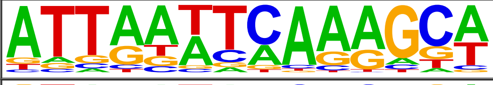	1e-15	-3.626e+01	0.21%	0.01%	58.7bp (65.3bp)	Tcf3(HMG)/mES-Tcf3-ChIP-Seq(GSE11724)/Homer(0.696) More Information | Similar Motifs Found	motif file (matrix)
18 *	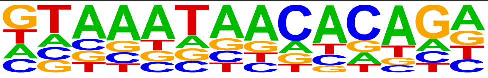	1e-10	-2.356e+01	0.13%	0.01%	43.8bp (35.6bp)	PB0119.1_Foxa2_2/Jaspar(0.749) More Information | Similar Motifs Found	motif file (matrix)
19 *	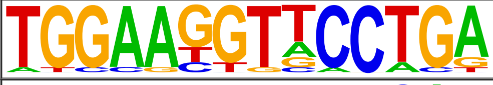	1e-7	-1.829e+01	0.15%	0.01%	60.6bp (75.3bp)	HRE(HSF)/HepG2-HSF1-ChIP-Seq(GSE31477)/Homer(0.739) More Information | Similar Motifs Found	motif file (matrix)
20 *	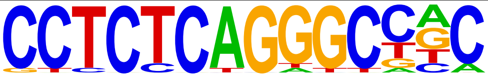	1e-7	-1.709e+01	0.12%	0.01%	59.3bp (44.6bp)	GLIS3(Zf)/Thyroid-Glis3.GFP-ChIP-Seq(GSE103297)/Homer(0.644) More Information | Similar Motifs Found	motif file (matrix)

Homer *de novo* Motif Results (RXR/)

Known Motif Enrichment Results
Gene Ontology Enrichment Results
If Homer is having trouble matching a motif to a known motif, try copy/pasting the matrix file into STAMP
More information on motif finding results: HOMER | Description of Results | Tips
Total target sequences = 7744
Total background sequences = 41985
* - possible false positive

Rank	Motif	P-value	log P-pvalue	% of Targets	% of Background	STD(Bg STD)	Best Match/Details	Motif File
1	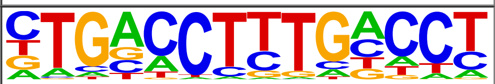	1e-641	-1.478e+03	35.33%	11.65%	51.4bp (60.4bp)	PPARa(NR),DR1/Liver-Ppara-ChIP-Seq(GSE47954)/Homer(0.974) More Information | Similar Motifs Found	motif file (matrix)
2	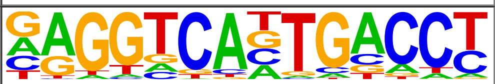	1e-322	-7.434e+02	11.57%	2.38%	47.8bp (60.2bp)	FXR(NR),IR1/Liver-FXR-ChIP-Seq(Chong_et_al.)/Homer(0.994) More Information | Similar Motifs Found	motif file (matrix)
3	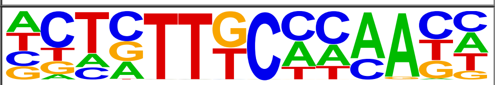	1e-154	-3.556e+02	19.33%	9.43%	52.9bp (58.6bp)	CEBPA/MA0102.3/Jaspar(0.870) More Information | Similar Motifs Found	motif file (matrix)
4	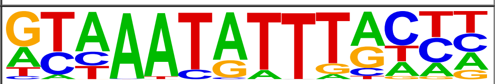	1e-76	-1.770e+02	3.03%	0.67%	54.4bp (59.7bp)	Arid5a/MA0602.1/Jaspar(0.774) More Information | Similar Motifs Found	motif file (matrix)
5	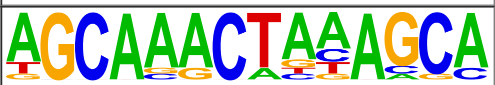	1e-33	-7.635e+01	0.26%	0.00%	54.3bp (54.4bp)	CEBP:CEBP(bZIP)/MEF-Chop-ChIP-Seq(GSE35681)/Homer(0.574) More Information | Similar Motifs Found	motif file (matrix)
6	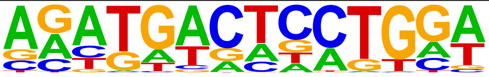	1e-33	-7.635e+01	0.26%	0.00%	51.0bp (0.0bp)	NFE2/MA0841.1/Jaspar(0.672) More Information | Similar Motifs Found	motif file (matrix)
7	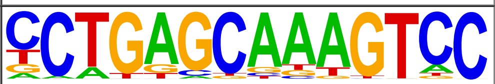	1e-28	-6.671e+01	0.43%	0.03%	57.2bp (57.3bp)	HNF4G/MA0484.1/Jaspar(0.687) More Information | Similar Motifs Found	motif file (matrix)
8	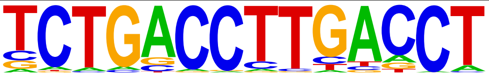	1e-23	-5.503e+01	1.14%	0.30%	46.2bp (67.4bp)	RARg(NR)/ES-RARg-ChIP-Seq(GSE30538)/Homer(0.907) More Information | Similar Motifs Found	motif file (matrix)
9	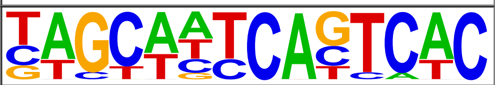	1e-23	-5.344e+01	0.19%	0.00%	47.5bp (47.0bp)	Pbx3(Homeobox)/GM12878-PBX3-ChIP-Seq(GSE32465)/Homer(0.672) More Information | Similar Motifs Found	motif file (matrix)
10	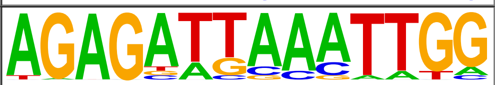	1e-22	-5.257e+01	0.36%	0.02%	48.4bp (58.5bp)	PH0124.1_Obox5_1/Jaspar(0.641) More Information | Similar Motifs Found	motif file (matrix)
11	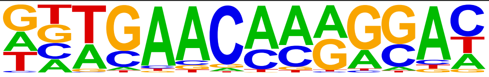	1e-19	-4.411e+01	0.22%	0.01%	43.6bp (0.0bp)	PB0061.1_Sox11_1/Jaspar(0.812) More Information | Similar Motifs Found	motif file (matrix)
12	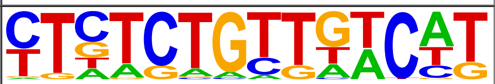	1e-17	-4.068e+01	0.21%	0.01%	45.4bp (35.9bp)	Foxo1(Forkhead)/RAW-Foxo1-ChIP-Seq(Fan_et_al.)/Homer(0.690) More Information | Similar Motifs Found	motif file (matrix)
13	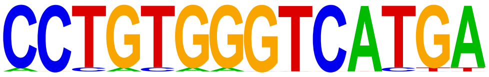	1e-16	-3.849e+01	0.25%	0.02%	42.2bp (61.5bp)	JUN::JUNB/MA1132.1/Jaspar(0.623) More Information | Similar Motifs Found	motif file (matrix)
14	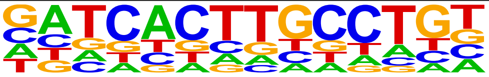	1e-15	-3.587e+01	0.17%	0.01%	42.3bp (18.7bp)	SD0002.1_at_AC_acceptor/Jaspar(0.849) More Information | Similar Motifs Found	motif file (matrix)
15	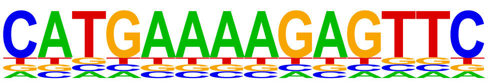	1e-15	-3.587e+01	0.17%	0.01%	38.3bp (82.1bp)	Hoxd10(Homeobox)/ChickenMSG-Hoxd10.Flag-ChIP-Seq(GSE86088)/Homer(0.573) More Information | Similar Motifs Found	motif file (matrix)
16	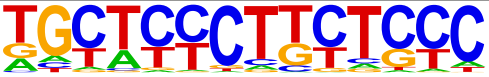	1e-13	-3.218e+01	0.13%	0.00%	44.5bp (28.6bp)	PB0137.1_Irf3_2/Jaspar(0.620) More Information | Similar Motifs Found	motif file (matrix)
17 *	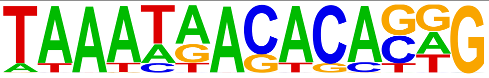	1e-11	-2.542e+01	0.13%	0.01%	43.3bp (31.2bp)	PB0119.1_Foxa2_2/Jaspar(0.662) More Information | Similar Motifs Found	motif file (matrix)
18 *	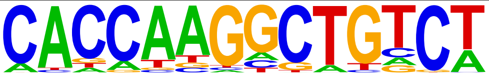	1e-10	-2.430e+01	0.10%	0.00%	51.8bp (0.0bp)	MEIS2/MA0774.1/Jaspar(0.577) More Information | Similar Motifs Found	motif file (matrix)

Homer Known Motif Enrichment Results (FXR)

Homer *de novo* Motif Results
Gene Ontology Enrichment Results
Known Motif Enrichment Results (txt file)
Total Target Sequences = 6697, Total Background Sequences = 42875

Rank	Motif	Name	P-value	log P-pvalue	q-value (Benjamini)	# Target Sequences with Motif	% of Targets Sequences with Motif	# Background Sequences with Motif	% of Background Sequences with Motif	Motif File	SVG
1	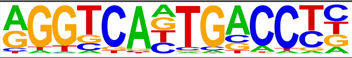	FXR(NR),IR1/Liver-FXR-ChIP-Seq(Chong_et_al.)/Homer	1e-898	-2.070e+03	0.0000	1857.0	27.73%	1886.5	4.40%	motif file (matrix)	svg
2	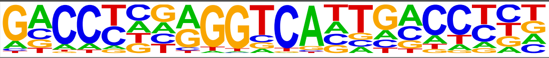	FXRa2(NR)_GSE133700,ER2/IR1-overlapping-motif_22nt(Ramos-Pittol_et_al.)	1e-768	-1.769e+03	0.0000	1361.0	20.32%	1081.6	2.52%	motif file (matrix)	svg
3	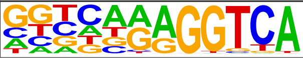	COUP-TFII(NR)/K562-NR2F1-ChIP-Seq(Encode)/Homer	1e-621	-1.432e+03	0.0000	3084.0	46.05%	7576.5	17.66%	motif file (matrix)	svg
4	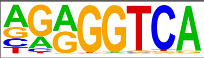	COUP-TFII(NR)/Artia-Nr2f2-ChIP-Seq(GSE46497)/Homer	1e-609	-1.404e+03	0.0000	3371.0	50.34%	9019.6	21.03%	motif file (matrix)	svg
5	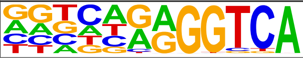	EAR2(NR)/K562-NR2F6-ChIP-Seq(Encode)/Homer	1e-588	-1.356e+03	0.0000	2917.0	43.56%	7075.2	16.50%	motif file (matrix)	svg
6	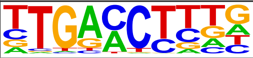	RARa(NR)/K562-RARa-ChIP-Seq(Encode)/Homer	1e-581	-1.339e+03	0.0000	4442.0	66.33%	15115.9	35.24%	motif file (matrix)	svg
7	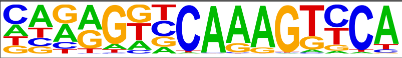	HNF4a(NR),DR1/HepG2-HNF4a-ChIP-Seq(GSE25021)/Homer	1e-475	-1.095e+03	0.0000	1476.0	22.04%	2268.7	5.29%	motif file (matrix)	svg
8	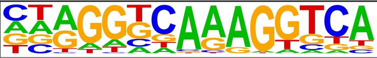	PPARa(NR),DR1/Liver-Ppara-ChIP-Seq(GSE47954)/Homer	1e-424	-9.778e+02	0.0000	2163.0	32.30%	5078.4	11.84%	motif file (matrix)	svg
9	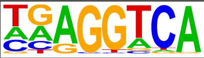	THRb(NR)/Liver-NR1A2-ChIP-Seq(GSE52613)/Homer	1e-416	-9.581e+02	0.0000	5080.0	75.85%	21360.7	49.80%	motif file (matrix)	svg
10	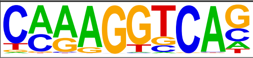	Erra(NR)/HepG2-Erra-ChIP-Seq(GSE31477)/Homer	1e-398	-9.166e+02	0.0000	3550.0	53.01%	12064.8	28.13%	motif file (matrix)	svg
11	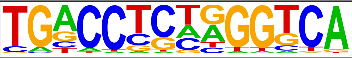	FXRa2(NR)_GSE133700,ER2-motif_15nt(Ramos-Pittol_et_al.)	1e-359	-8.278e+02	0.0000	1305.0	19.49%	2274.3	5.30%	motif file (matrix)	svg
12	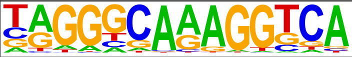	RXR(NR),DR1/3T3L1-RXR-ChIP-Seq(GSE13511)/Homer	1e-297	-6.855e+02	0.0000	1984.0	29.63%	5378.3	12.54%	motif file (matrix)	svg
13	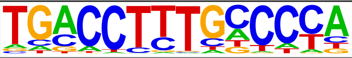	PPARE(NR),DR1/3T3L1-Pparg-ChIP-Seq(GSE13511)/Homer	1e-282	-6.509e+02	0.0000	1761.0	26.30%	4547.6	10.60%	motif file (matrix)	svg
14	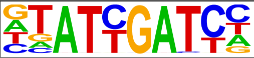	HNF6(Homeobox)/Liver-Hnf6-ChIP-Seq(ERP000394)/Homer	1e-232	-5.352e+02	0.0000	915.0	13.66%	1664.5	3.88%	motif file (matrix)	svg
15	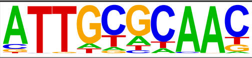	CEBP(bZIP)/ThioMac-CEBPb-ChIP-Seq(GSE21512)/Homer	1e-229	-5.296e+02	0.0000	1014.0	15.14%	2019.5	4.71%	motif file (matrix)	svg
16	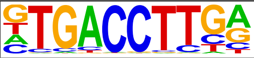	Esrrb(NR)/mES-Esrrb-ChIP-Seq(GSE11431)/Homer	1e-222	-5.115e+02	0.0000	1391.0	20.77%	3532.2	8.24%	motif file (matrix)	svg
17	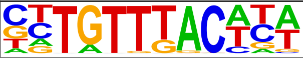	Foxa2(Forkhead)/Liver-Foxa2-ChIP-Seq(GSE25694)/Homer	1e-194	-4.480e+02	0.0000	1172.0	17.50%	2879.1	6.71%	motif file (matrix)	svg
18	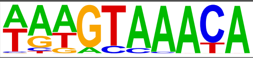	FOXA1(Forkhead)/LNCAP-FOXA1-ChIP-Seq(GSE27824)/Homer	1e-183	-4.226e+02	0.0000	1467.0	21.91%	4239.5	9.88%	motif file (matrix)	svg
19	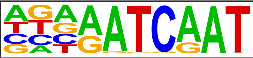	Cux2(Homeobox)/Liver-Cux2-ChIP-Seq(GSE35985)/Homer	1e-179	-4.125e+02	0.0000	709.0	10.59%	1281.5	2.99%	motif file (matrix)	svg
20	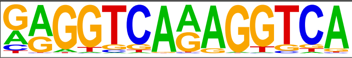	TR4(NR),DR1/Hela-TR4-ChIP-Seq(GSE24685)/Homer	1e-177	-4.095e+02	0.0000	482.0	7.20%	611.1	1.42%	motif file (matrix)	svg
21	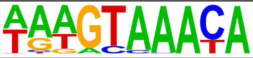	FOXA1(Forkhead)/MCF7-FOXA1-ChIP-Seq(GSE26831)/Homer	1e-169	-3.893e+02	0.0000	1217.0	18.17%	3302.0	7.70%	motif file (matrix)	svg
22	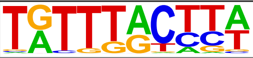	FOXM1(Forkhead)/MCF7-FOXM1-ChIP-Seq(GSE72977)/Homer	1e-160	-3.694e+02	0.0000	1305.0	19.49%	3774.4	8.80%	motif file (matrix)	svg
23	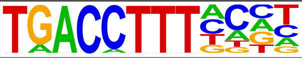	Nur77(NR)/K562-NR4A1-ChIP-Seq(GSE31363)/Homer	1e-155	-3.577e+02	0.0000	480.0	7.17%	693.5	1.62%	motif file (matrix)	svg
24	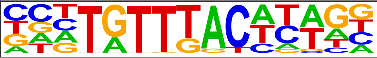	Foxa3(Forkhead)/Liver-Foxa3-ChIP-Seq(GSE77670)/Homer	1e-153	-3.524e+02	0.0000	574.0	8.57%	987.7	2.30%	motif file (matrix)	svg
25	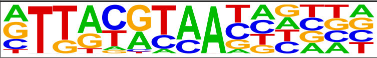	NFIL3(bZIP)/HepG2-NFIL3-ChIP-Seq(Encode)/Homer	1e-147	-3.385e+02	0.0000	746.0	11.14%	1615.8	3.77%	motif file (matrix)	svg
26	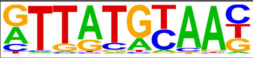	HLF(bZIP)/HSC-HLF.Flag-ChIP-Seq(GSE69817)/Homer	1e-140	-3.227e+02	0.0000	932.0	13.92%	2393.0	5.58%	motif file (matrix)	svg
27	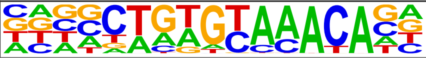	Fox:Ebox(Forkhead,bHLH)/Panc1-Foxa2-ChIP-Seq(GSE47459)/Homer	1e-137	-3.158e+02	0.0000	1369.0	20.44%	4344.0	10.13%	motif file (matrix)	svg
28	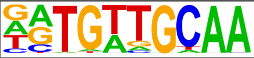	CEBP:AP1(bZIP)/ThioMac-CEBPb-ChIP-Seq(GSE21512)/Homer	1e-98	-2.260e+02	0.0000	866.0	12.93%	2553.8	5.95%	motif file (matrix)	svg
29	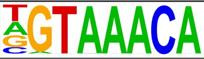	Foxo3(Forkhead)/U2OS-Foxo3-ChIP-Seq(E-MTAB-2701)/Homer	1e-94	-2.185e+02	0.0000	825.0	12.32%	2412.7	5.63%	motif file (matrix)	svg
30	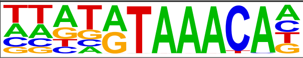	Foxf1(Forkhead)/Lung-Foxf1-ChIP-Seq(GSE77951)/Homer	1e-91	-2.097e+02	0.0000	883.0	13.19%	2715.5	6.33%	motif file (matrix)	svg
31	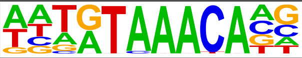	FoxL2(Forkhead)/Ovary-FoxL2-ChIP-Seq(GSE60858)/Homer	1e-90	-2.092e+02	0.0000	858.0	12.81%	2606.2	6.08%	motif file (matrix)	svg
32	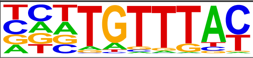	FOXK1(Forkhead)/HEK293-FOXK1-ChIP-Seq(GSE51673)/Homer	1e-90	-2.074e+02	0.0000	1051.0	15.69%	3502.4	8.17%	motif file (matrix)	svg

Homer Known Motif Enrichment Results (RXR)

Homer *de novo* Motif Results
Gene Ontology Enrichment Results
Known Motif Enrichment Results (txt file)
Total Target Sequences = 7744, Total Background Sequences = 41983

Rank	Motif	Name	P-value	log P-pvalue	q-value (Benjamini)	# Target Sequences with Motif	% of Targets Sequences with Motif	# Background Sequences with Motif	% of Background Sequences with Motif	Motif File	SVG
1	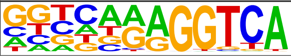	COUP-TFII(NR)/K562-NR2F1-ChIP-Seq(Encode)/Homer	1e-640	-1.474e+03	0.0000	3382.0	43.67%	7225.3	17.21%	motif file (matrix)	svg
2	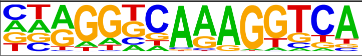	PPARa(NR),DR1/Liver-Ppara-ChIP-Seq(GSE47954)/Homer	1e-611	-1.408e+03	0.0000	2746.0	35.46%	5094.6	12.13%	motif file (matrix)	svg
3	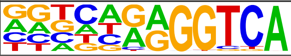	EAR2(NR)/K562-NR2F6-ChIP-Seq(Encode)/Homer	1e-570	-1.314e+03	0.0000	3129.0	40.41%	6732.8	16.03%	motif file (matrix)	svg
4	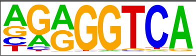	COUP-TFII(NR)/Artia-Nr2f2-ChIP-Seq(GSE46497)/Homer	1e-555	-1.279e+03	0.0000	3556.0	45.92%	8538.1	20.33%	motif file (matrix)	svg
5	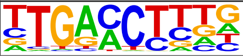	RARa(NR)/K562-RARa-ChIP-Seq(Encode)/Homer	1e-544	-1.254e+03	0.0000	4942.0	63.82%	15036.6	35.81%	motif file (matrix)	svg
6	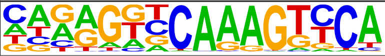	HNF4a(NR),DR1/HepG2-HNF4a-ChIP-Seq(GSE25021)/Homer	1e-532	-1.227e+03	0.0000	1705.0	22.02%	2279.1	5.43%	motif file (matrix)	svg
7	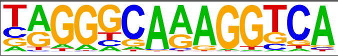	RXR(NR),DR1/3T3L1-RXR-ChIP-Seq(GSE13511)/Homer	1e-486	-1.121e+03	0.0000	2543.0	32.84%	5145.5	12.25%	motif file (matrix)	svg
8	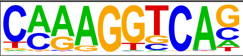	Erra(NR)/HepG2-Erra-ChIP-Seq(GSE31477)/Homer	1e-452	-1.042e+03	0.0000	4005.0	51.72%	11425.1	27.21%	motif file (matrix)	svg
9	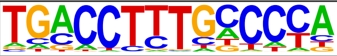	PPARE(NR),DR1/3T3L1-Pparg-ChIP-Seq(GSE13511)/Homer	1e-451	-1.041e+03	0.0000	2281.0	29.46%	4453.1	10.60%	motif file (matrix)	svg
10	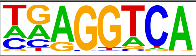	THRb(NR)/Liver-NR1A2-ChIP-Seq(GSE52613)/Homer	1e-387	-8.925e+02	0.0000	5659.0	73.08%	20812.9	49.57%	motif file (matrix)	svg
11	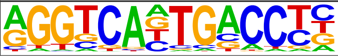	FXR(NR),IR1/Liver-FXR-ChIP-Seq(Chong_et_al.)/Homer	1e-316	-7.297e+02	0.0000	1171.0	15.12%	1722.8	4.10%	motif file (matrix)	svg
12	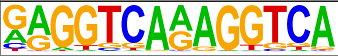	TR4(NR),DR1/Hela-TR4-ChIP-Seq(GSE24685)/Homer	1e-281	-6.489e+02	0.0000	656.0	8.47%	597.6	1.42%	motif file (matrix)	svg
13	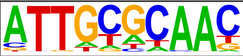	CEBP(bZIP)/ThioMac-CEBPb-ChIP-Seq(GSE21512)/Homer	1e-270	-6.240e+02	0.0000	1216.0	15.70%	2079.4	4.95%	motif file (matrix)	svg
14	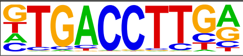	Esrrb(NR)/mES-Esrrb-ChIP-Seq(GSE11431)/Homer	1e-261	-6.015e+02	0.0000	1558.0	20.12%	3249.9	7.74%	motif file (matrix)	svg
15	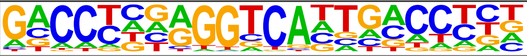	FXRa2(NR)_GSE133700,ER2/IR1-overlapping-motif_22nt(Ramos-Pittol_et_al.)	1e-247	-5.692e+02	0.0000	736.0	9.50%	874.7	2.08%	motif file (matrix)	svg
16	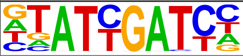	HNF6(Homeobox)/Liver-Hnf6-ChIP-Seq(ERP000394)/Homer	1e-227	-5.248e+02	0.0000	1025.0	13.24%	1741.0	4.15%	motif file (matrix)	svg
17	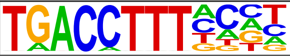	Nur77(NR)/K562-NR4A1-ChIP-Seq(GSE31363)/Homer	1e-211	-4.880e+02	0.0000	604.0	7.80%	683.3	1.63%	motif file (matrix)	svg
18	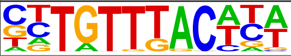	Foxa2(Forkhead)/Liver-Foxa2-ChIP-Seq(GSE25694)/Homer	1e-196	-4.513e+02	0.0000	1348.0	17.41%	3014.4	7.18%	motif file (matrix)	svg
19	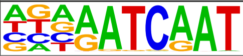	Cux2(Homeobox)/Liver-Cux2-ChIP-Seq(GSE35985)/Homer	1e-192	-4.425e+02	0.0000	835.0	10.78%	1371.4	3.27%	motif file (matrix)	svg
20	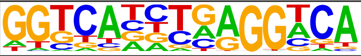	THRa(NR)/C17.2-THRa-ChIP-Seq(GSE38347)/Homer	1e-183	-4.215e+02	0.0000	975.0	12.59%	1851.8	4.41%	motif file (matrix)	svg
21	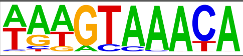	FOXA1(Forkhead)/LNCAP-FOXA1-ChIP-Seq(GSE27824)/Homer	1e-169	-3.911e+02	0.0000	1711.0	22.09%	4633.7	11.03%	motif file (matrix)	svg
22	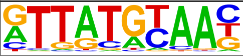	HLF(bZIP)/HSC-HLF.Flag-ChIP-Seq(GSE69817)/Homer	1e-167	-3.856e+02	0.0000	1146.0	14.80%	2535.7	6.04%	motif file (matrix)	svg
23	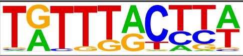	FOXM1(Forkhead)/MCF7-FOXM1-ChIP-Seq(GSE72977)/Homer	1e-165	-3.801e+02	0.0000	1543.0	19.93%	4024.0	9.58%	motif file (matrix)	svg
24	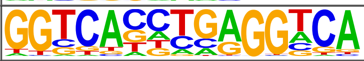	THRb(NR)/HepG2-THRb.Flag-ChIP-Seq(Encode)/Homer	1e-164	-3.779e+02	0.0000	1138.0	14.70%	2534.2	6.04%	motif file (matrix)	svg
25	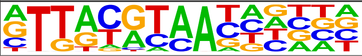	NFIL3(bZIP)/HepG2-NFIL3-ChIP-Seq(Encode)/Homer	1e-156	-3.601e+02	0.0000	900.0	11.62%	1787.9	4.26%	motif file (matrix)	svg
26	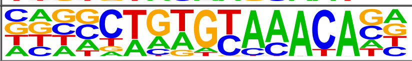	Fox:Ebox(Forkhead,bHLH)/Panc1-Foxa2-ChIP-Seq(GSE47459)/Homer	1e-151	-3.477e+02	0.0000	1585.0	20.47%	4343.3	10.34%	motif file (matrix)	svg
27	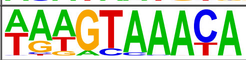	FOXA1(Forkhead)/MCF7-FOXA1-ChIP-Seq(GSE26831)/Homer	1e-149	-3.453e+02	0.0000	1414.0	18.26%	3684.3	8.77%	motif file (matrix)	svg
28	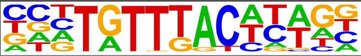	Foxa3(Forkhead)/Liver-Foxa3-ChIP-Seq(GSE77670)/Homer	1e-141	-3.255e+02	0.0000	645.0	8.33%	1089.2	2.59%	motif file (matrix)	svg
29	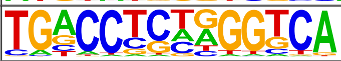	FXRa2(NR)_GSE133700,ER2-motif_15nt(Ramos-Pittol_et_al.)	1e-105	-2.424e+02	0.0000	874.0	11.29%	2111.0	5.03%	motif file (matrix)	svg
30	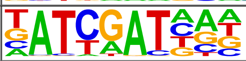	CUX1(Homeobox)/K562-CUX1-ChIP-Seq(GSE92882)/Homer	1e-95	-2.190e+02	0.0000	849.0	10.96%	2118.4	5.04%	motif file (matrix)	svg
31	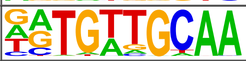	CEBP:AP1(bZIP)/ThioMac-CEBPb-ChIP-Seq(GSE21512)/Homer	1e-94	-2.187e+02	0.0000	995.0	12.85%	2671.8	6.36%	motif file (matrix)	svg
32	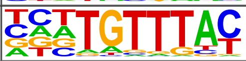	FOXK1(Forkhead)/HEK293-FOXK1-ChIP-Seq(GSE51673)/Homer	1e-90	-2.084e+02	0.0000	1265.0	16.34%	3809.1	9.07%	motif file (matrix)	svg
33	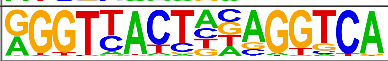	LXRE(NR),DR4/RAW-LXRb.biotin-ChIP-Seq(GSE21512)/Homer	1e-88	-2.030e+02	0.0000	204.0	2.63%	182.2	0.43%	motif file (matrix)	svg
34	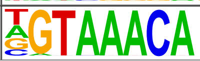	Foxo3(Forkhead)/U2OS-Foxo3-ChIP-Seq(E-MTAB-2701)/Homer	1e-80	-1.861e+02	0.0000	960.0	12.40%	2704.9	6.44%	motif file (matrix)	svg

Homer Known Motif Enrichment Results (FXR-RXRproximal)

[Homer *de novo* Motif Results](#)
[Gene Ontology Enrichment Results](#)
[Known Motif Enrichment Results (txt file)](#)
Total Target Sequences = 4435, Total Background Sequences = 44868

Rank	Motif	Name	P-value	log P-pvalue	q-value (Benjamini)	# Target Sequences with Motif	% of Targets Sequences with Motif	# Background Sequences with Motif	% of Background Sequences with Motif	Motif File	SVG
1	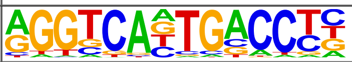	FXR(NR),IR1/Liver-FXR-ChIP-Seq(Chong_et_al.)/Homer	1e-621	-1.431e+03	0.0000	1239.0	27.94%	1902.4	4.24%	motif file (matrix)	svg
2	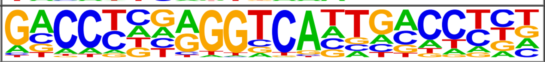	FXRa2(NR)_GSE133700,ER2/IR1-overlapping-motif_22nt(Ramos-Pittol_et_al.)	1e-497	-1.146e+03	0.0000	868.0	19.58%	1062.5	2.37%	motif file (matrix)	svg
3	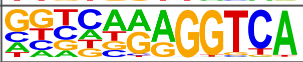	COUP-TFII(NR)/K562-NR2F1-ChIP-Seq(Encode)/Homer	1e-470	-1.083e+03	0.0000	2150.0	48.49%	8022.0	17.89%	motif file (matrix)	svg
4	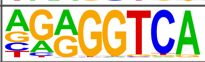	COUP-TFII(NR)/Artia-Nr2f2-ChIP-Seq(GSE46497)/Homer	1e-435	-1.003e+03	0.0000	2296.0	51.78%	9512.7	21.21%	motif file (matrix)	svg
5	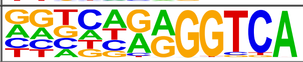	EAR2(NR)/K562-NR2F6-ChIP-Seq(Encode)/Homer	1e-427	-9.838e+02	0.0000	2015.0	45.44%	7556.5	16.85%	motif file (matrix)	svg
6	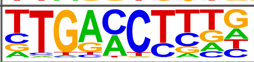	RARa(NR)/K562-RARa-ChIP-Seq(Encode)/Homer	1e-425	-9.798e+02	0.0000	3049.0	68.76%	16171.1	36.06%	motif file (matrix)	svg
7	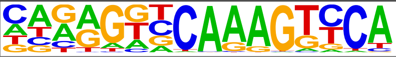	HNF4a(NR),DR1/HepG2-HNF4a-ChIP-Seq(GSE25021)/Homer	1e-407	-9.387e+02	0.0000	1101.0	24.83%	2366.1	5.28%	motif file (matrix)	svg
8	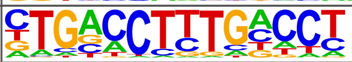	1-CTGACCTTTGACCT,DR1/RXRAmotif1	1e-361	-8.322e+02	0.0000	1591.0	35.88%	5446.8	12.15%	motif file (matrix)	svg
9	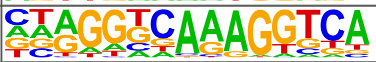	PPARa(NR),DR1/Liver-Ppara-ChIP-Seq(GSE47954)/Homer	1e-355	-8.190e+02	0.0000	1577.0	35.57%	5417.9	12.08%	motif file (matrix)	svg
10	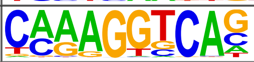	Erra(NR)/HepG2-Erra-ChIP-Seq(GSE31477)/Homer	1e-310	-7.157e+02	0.0000	2466.0	55.62%	12769.6	28.48%	motif file (matrix)	svg
11	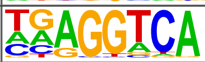	THRb(NR)/Liver-NR1A2-ChIP-Seq(GSE52613)/Homer	1e-309	-7.116e+02	0.0000	3467.0	78.19%	22786.3	50.82%	motif file (matrix)	svg
12	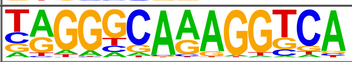	RXR(NR),DR1/3T3L1-RXR-ChIP-Seq(GSE13511)/Homer	1e-247	-5.690e+02	0.0000	1433.0	32.32%	5750.5	12.82%	motif file (matrix)	svg
13	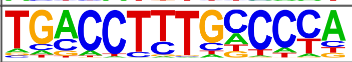	PPARE(NR),DR1/3T3L1-Pparg-ChIP-Seq(GSE13511)/Homer	1e-226	-5.225e+02	0.0000	1263.0	28.48%	4874.9	10.87%	motif file (matrix)	svg
14	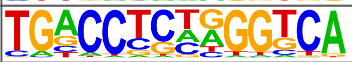	FXRa2(NR)_GSE133700,ER2-motif_15nt(Ramos-Pittol_et_al.)	1e-212	-4.885e+02	0.0000	821.0	18.52%	2374.0	5.29%	motif file (matrix)	svg
15	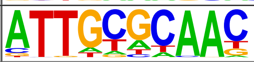	CEBP(bZIP)/ThioMac-CEBPb-ChIP-Seq(GSE21512)/Homer	1e-195	-4.504e+02	0.0000	748.0	16.87%	2129.6	4.75%	motif file (matrix)	svg
16	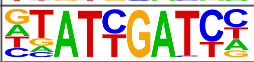	HNF6(Homeobox)/Liver-Hnf6-ChIP-Seq(ERP000394)/Homer	1e-190	-4.391e+02	0.0000	652.0	14.70%	1677.7	3.74%	motif file (matrix)	svg
17	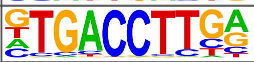	Esrrb(NR)/mES-Esrrb-ChIP-Seq(GSE11431)/Homer	1e-176	-4.065e+02	0.0000	985.0	22.21%	3719.8	8.30%	motif file (matrix)	svg
18	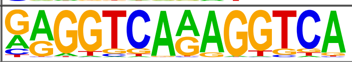	TR4(NR),DR1/Hela-TR4-ChIP-Seq(GSE24685)/Homer	1e-172	-3.964e+02	0.0000	383.0	8.64%	619.5	1.38%	motif file (matrix)	svg
19	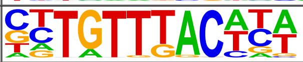	Foxa2(Forkhead)/Liver-Foxa2-ChIP-Seq(GSE25694)/Homer	1e-147	-3.395e+02	0.0000	819.0	18.47%	3044.7	6.79%	motif file (matrix)	svg
20	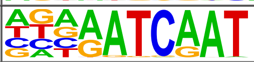	Cux2(Homeobox)/Liver-Cux2-ChIP-Seq(GSE35985)/Homer	1e-146	-3.370e+02	0.0000	505.0	11.39%	1294.7	2.89%	motif file (matrix)	svg
21	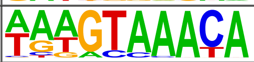	FOXA1(Forkhead)/LNCAP-FOXA1-ChIP-Seq(GSE27824)/Homer	1e-145	-3.345e+02	0.0000	1050.0	23.68%	4596.4	10.25%	motif file (matrix)	svg
22	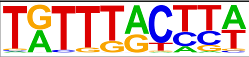	FOXM1(Forkhead)/MCF7-FOXM1-ChIP-Seq(GSE72977)/Homer	1e-137	-3.168e+02	0.0000	946.0	21.34%	4005.3	8.93%	motif file (matrix)	svg
23	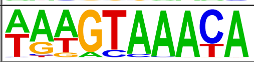	FOXA1(Forkhead)/MCF7-FOXA1-ChIP-Seq(GSE26831)/Homer	1e-132	-3.041e+02	0.0000	864.0	19.49%	3541.2	7.90%	motif file (matrix)	svg
24	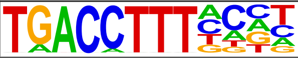	Nur77(NR)/K562-NR4A1-ChIP-Seq(GSE31363)/Homer	1e-125	-2.883e+02	0.0000	347.0	7.83%	715.3	1.60%	motif file (matrix)	svg
25	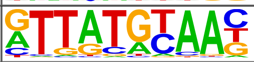	HLF(bZIP)/HSC-HLF.Flag-ChIP-Seq(GSE69817)/Homer	1e-121	-2.791e+02	0.0000	682.0	15.38%	2529.1	5.64%	motif file (matrix)	svg
26	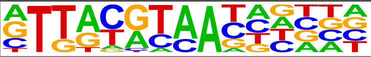	NFIL3(bZIP)/HepG2-NFIL3-ChIP-Seq(Encode)/Homer	1e-119	-2.751e+02	0.0000	544.0	12.27%	1738.0	3.88%	motif file (matrix)	svg
27	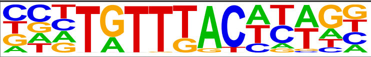	Foxa3(Forkhead)/Liver-Foxa3-ChIP-Seq(GSE77670)/Homer	1e-118	-2.729e+02	0.0000	416.0	9.38%	1075.4	2.40%	motif file (matrix)	svg
28	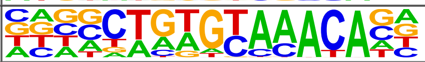	Fox:Ebox(Forkhead,bHLH)/Panc1-Foxa2-ChIP-Seq(GSE47459)/Homer	1e-115	-2.668e+02	0.0000	984.0	22.19%	4631.4	10.33%	motif file (matrix)	svg
29	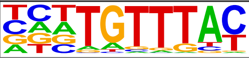	FOXK1(Forkhead)/HEK293-FOXK1-ChIP-Seq(GSE51673)/Homer	1e-80	-1.850e+02	0.0000	761.0	17.16%	3705.5	8.26%	motif file (matrix)	svg
30	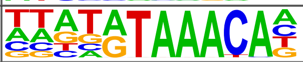	Foxf1(Forkhead)/Lung-Foxf1-ChIP-Seq(GSE77951)/Homer	1e-74	-1.707e+02	0.0000	626.0	14.12%	2873.6	6.41%	motif file (matrix)	svg
31	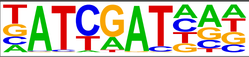	CUX1(Homeobox)/K562-CUX1-ChIP-Seq(GSE92882)/Homer	1e-73	-1.697e+02	0.0000	489.0	11.03%	1971.3	4.40%	motif file (matrix)	svg
32	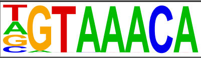	Foxo3(Forkhead)/U2OS-Foxo3-ChIP-Seq(E-MTAB-2701)/Homer	1e-73	-1.693e+02	0.0000	583.0	13.15%	2590.8	5.78%	motif file (matrix)	svg
33	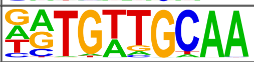	CEBP:AP1(bZIP)/ThioMac-CEBPb-ChIP-Seq(GSE21512)/Homer	1e-70	-1.619e+02	0.0000	598.0	13.49%	2749.4	6.13%	motif file (matrix)	svg

Homer Known Motif Enrichment Results (FXR-RXRdistal)

Homer *de novo* Motif Results
Gene Ontology Enrichment Results
Known Motif Enrichment Results (txt file)
Total Target Sequences = 2263, Total Background Sequences = 47332

Rank	Motif	Name	P-value	log P-pvalue	q-value (Benjamini)	# Target Sequences with Motif	% of Targets Sequences with Motif	# Background Sequences with Motif	% of Background Sequences with Motif	Motif File	SVG
1	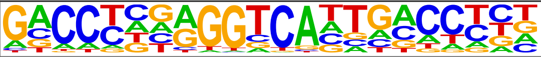	FXRa2(NR)_GSE133700,ER2/IR1-overlapping-motif_22nt(Ramos-Pittol_et_al.)	1e-473	-1.089e+03	0.0000	635.0	28.06%	1098.1	2.32%	motif file (matrix)	svg
2	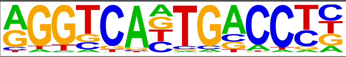	FXR(NR),IR1/Liver-FXR-ChIP-Seq(Chong_et_al.)/Homer	1e-432	-9.955e+02	0.0000	746.0	32.97%	2000.6	4.22%	motif file (matrix)	svg
3	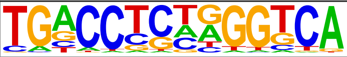	FXRa2(NR)_GSE133700,ER2-motif_15nt(Ramos-Pittol_et_al.)	1e-223	-5.157e+02	0.0000	565.0	24.97%	2348.6	4.96%	motif file (matrix)	svg
4	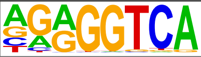	COUP-TFII(NR)/Artia-Nr2f2-ChIP-Seq(GSE46497)/Homer	1e-203	-4.676e+02	0.0000	1117.0	49.36%	9718.0	20.52%	motif file (matrix)	svg
5	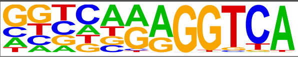	COUP-TFII(NR)/K562-NR2F1-ChIP-Seq(Encode)/Homer	1e-197	-4.555e+02	0.0000	1003.0	44.32%	8119.7	17.15%	motif file (matrix)	svg
6	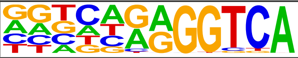	EAR2(NR)/K562-NR2F6-ChIP-Seq(Encode)/Homer	1e-186	-4.302e+02	0.0000	948.0	41.89%	7592.7	16.03%	motif file (matrix)	svg
7	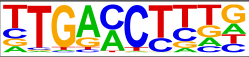	RARa(NR)/K562-RARa-ChIP-Seq(Encode)/Homer	1e-177	-4.077e+02	0.0000	1457.0	64.38%	16561.7	34.97%	motif file (matrix)	svg
8	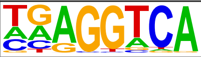	THRb(NR)/Liver-NR1A2-ChIP-Seq(GSE52613)/Homer	1e-142	-3.282e+02	0.0000	1690.0	74.68%	22927.4	48.42%	motif file (matrix)	svg
9	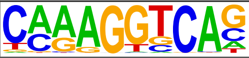	Erra(NR)/HepG2-Erra-ChIP-Seq(GSE31477)/Homer	1e-128	-2.968e+02	0.0000	1162.0	51.35%	12905.8	27.25%	motif file (matrix)	svg
10	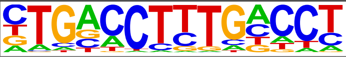	1-CTGACCTTTGACCT,DR1/RXRAmotif1	1e-115	-2.662e+02	0.0000	655.0	28.94%	5314.9	11.22%	motif file (matrix)	svg
11	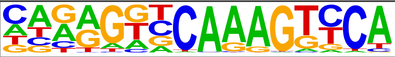	HNF4a(NR),DR1/HepG2-HNF4a-ChIP-Seq(GSE25021)/Homer	1e-111	-2.565e+02	0.0000	415.0	18.34%	2420.0	5.11%	motif file (matrix)	svg
12	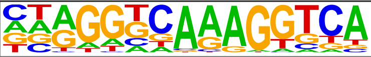	PPARa(NR),DR1/Liver-Ppara-ChIP-Seq(GSE47954)/Homer	1e-103	-2.375e+02	0.0000	636.0	28.10%	5399.1	11.40%	motif file (matrix)	svg
13	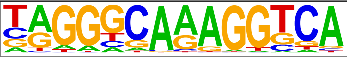	RXR(NR),DR1/3T3L1-RXR-ChIP-Seq(GSE13511)/Homer	1e-70	-1.620e+02	0.0000	579.0	25.59%	5641.3	11.91%	motif file (matrix)	svg
14	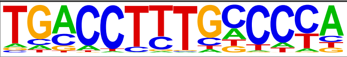	PPARE(NR),DR1/3T3L1-Pparg-ChIP-Seq(GSE13511)/Homer	1e-68	-1.583e+02	0.0000	516.0	22.80%	4771.1	10.08%	motif file (matrix)	svg
15	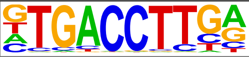	Esrrb(NR)/mES-Esrrb-ChIP-Seq(GSE11431)/Homer	1e-68	-1.570e+02	0.0000	439.0	19.40%	3709.5	7.83%	motif file (matrix)	svg
16	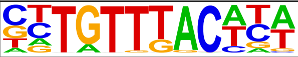	Foxa2(Forkhead)/Liver-Foxa2-ChIP-Seq(GSE25694)/Homer	1e-51	-1.182e+02	0.0000	361.0	15.95%	3168.6	6.69%	motif file (matrix)	svg
17	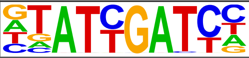	HNF6(Homeobox)/Liver-Hnf6-ChIP-Seq(ERP000394)/Homer	1e-51	-1.178e+02	0.0000	264.0	11.67%	1912.6	4.04%	motif file (matrix)	svg
18	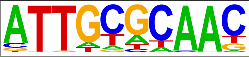	CEBP(bZIP)/ThioMac-CEBPb-ChIP-Seq(GSE21512)/Homer	1e-50	-1.166e+02	0.0000	298.0	13.17%	2351.9	4.97%	motif file (matrix)	svg
19	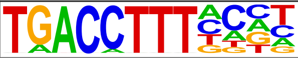	Nur77(NR)/K562-NR4A1-ChIP-Seq(GSE31363)/Homer	1e-47	-1.084e+02	0.0000	146.0	6.45%	701.1	1.48%	motif file (matrix)	svg
20	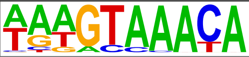	FOXA1(Forkhead)/LNCAP-FOXA1-ChIP-Seq(GSE27824)/Homer	1e-43	-9.928e+01	0.0000	455.0	20.11%	4858.4	10.26%	motif file (matrix)	svg
21	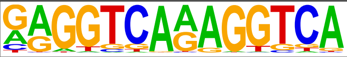	TR4(NR),DR1/Hela-TR4-ChIP-Seq(GSE24685)/Homer	1e-41	-9.604e+01	0.0000	125.0	5.52%	581.8	1.23%	motif file (matrix)	svg
22	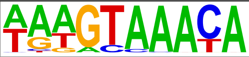	FOXA1(Forkhead)/MCF7-FOXA1-ChIP-Seq(GSE26831)/Homer	1e-41	-9.541e+01	0.0000	385.0	17.01%	3870.6	8.17%	motif file (matrix)	svg
23	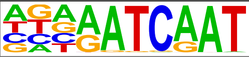	Cux2(Homeobox)/Liver-Cux2-ChIP-Seq(GSE35985)/Homer	1e-38	-8.802e+01	0.0000	201.0	8.88%	1466.5	3.10%	motif file (matrix)	svg
24	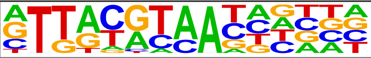	NFIL3(bZIP)/HepG2-NFIL3-ChIP-Seq(Encode)/Homer	1e-38	-8.792e+01	0.0000	242.0	10.69%	1983.2	4.19%	motif file (matrix)	svg
25	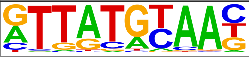	HLF(bZIP)/HSC-HLF.Flag-ChIP-Seq(GSE69817)/Homer	1e-37	-8.641e+01	0.0000	296.0	13.08%	2735.6	5.78%	motif file (matrix)	svg
26	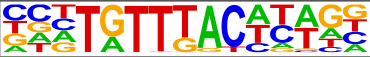	Foxa3(Forkhead)/Liver-Foxa3-ChIP-Seq(GSE77670)/Homer	1e-36	-8.462e+01	0.0000	172.0	7.60%	1158.6	2.45%	motif file (matrix)	svg
27	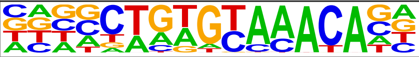	Fox:Ebox(Forkhead,bHLH)/Panc1-Foxa2-ChIP-Seq(GSE47459)/Homer	1e-35	-8.124e+01	0.0000	419.0	18.52%	4665.8	9.85%	motif file (matrix)	svg
28	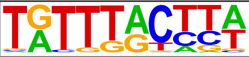	FOXM1(Forkhead)/MCF7-FOXM1-ChIP-Seq(GSE72977)/Homer	1e-32	-7.567e+01	0.0000	389.0	17.19%	4314.7	9.11%	motif file (matrix)	svg
29	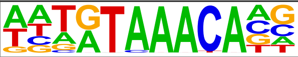	FoxL2(Forkhead)/Ovary-FoxL2-ChIP-Seq(GSE60858)/Homer	1e-24	-5.577e+01	0.0000	280.0	12.37%	3053.9	6.45%	motif file (matrix)	svg
30	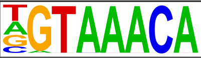	Foxo3(Forkhead)/U2OS-Foxo3-ChIP-Seq(E-MTAB-2701)/Homer	1e-23	-5.414e+01	0.0000	259.0	11.44%	2768.9	5.85%	motif file (matrix)	svg
31	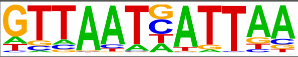	HNF1b(Homeobox)/PDAC-HNF1B-ChIP-Seq(GSE64557)/Homer	1e-23	-5.378e+01	0.0000	72.0	3.18%	347.2	0.73%	motif file (matrix)	svg
32	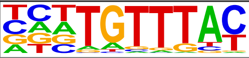	FOXK1(Forkhead)/HEK293-FOXK1-ChIP-Seq(GSE51673)/Homer	1e-22	-5.225e+01	0.0000	334.0	14.76%	3983.9	8.41%	motif file (matrix)	svg
33	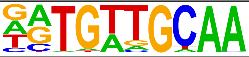	CEBP:AP1(bZIP)/ThioMac-CEBPb-ChIP-Seq(GSE21512)/Homer	1e-22	-5.100e+01	0.0000	257.0	11.36%	2803.0	5.92%	motif file (matrix)	svg
