# Supplementary material for: The farnesoid X receptor activates transcription independently of RXR at non-canonical response elements
Source: Nucleic Acids Res. 2024 Dec 9;53(4):gkae1214. doi: 10.1093/nar/gkae1214 (PMC11879013; doi:10.1093/nar/gkae1214)
Supplement: gkae1214_Supplemental_Files [file gkae1214_supplemental_files.zip › SD7-FXRa2 target promoters motif analysis.pdf]

Homer Known Motif Enrichment Results (FXRa2wt_up)

Homer *de novo* Motif Results
Gene Ontology Enrichment Results
Known Motif Enrichment Results (txt file)
Total Target Sequences = 1395, Total Background Sequences = 44753

Rank	Motif	Name	P-value	log P-pvalue	q-value (Benjamini)	# Target Sequences with Motif	% of Targets Sequences with Motif	# Background Sequences with Motif	% of Background Sequences with Motif	Motif File	SVG
1	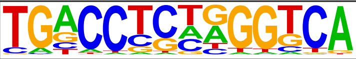	FXRa2(NR)_GSE133700,ER2-motif_15nt(Ramos-Pittol_et_al.)	1e-4	-1.059e+01	0.0105	135.0	9.68%	3007.4	6.72%	motif file (matrix)	svg
2	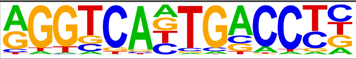	FXR(NR),IR1/Liver-FXR-ChIP-Seq(Chong_et_al.)/Homer	1e-4	-9.761e+00	0.0120	117.0	8.39%	2577.2	5.76%	motif file (matrix)	svg
3	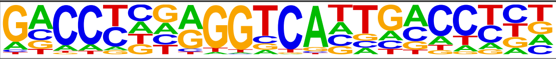	FXRa2(NR)_GSE133700,ER2/IR1-overlapping-motif_22nt(Ramos-Pittol_et_al.)	1e-3	-8.895e+00	0.0191	85.0	6.09%	1783.7	3.99%	motif file (matrix)	svg
4	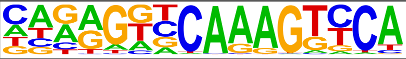	HNF4a(NR),DR1/HepG2-HNF4a-ChIP-Seq(GSE25021)/Homer	1e-3	-8.362e+00	0.0244	96.0	6.88%	2106.8	4.71%	motif file (matrix)	svg
5	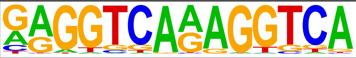	TR4(NR),DR1/Hela-TR4-ChIP-Seq(GSE24685)/Homer	1e-2	-6.437e+00	0.1335	38.0	2.72%	713.9	1.59%	motif file (matrix)	svg
6	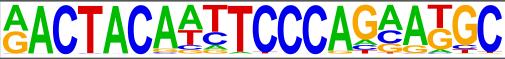	GFY-Staf(?,Zf)/Promoter/Homer	1e-2	-5.406e+00	0.3120	76.0	5.45%	1772.5	3.96%	motif file (matrix)	svg
7	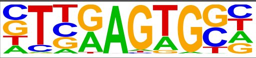	Nkx2.2(Homeobox)/NPC-Nkx2.2-ChIP-Seq(GSE61673)/Homer	1e-2	-5.278e+00	0.3120	353.0	25.30%	9990.2	22.32%	motif file (matrix)	svg
8	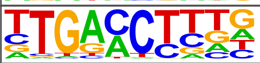	RARa(NR)/K562-RARa-ChIP-Seq(Encode)/Homer	1e-2	-5.159e+00	0.3120	430.0	30.82%	12390.8	27.68%	motif file (matrix)	svg
9	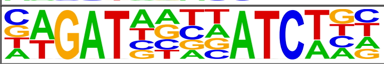	GATA(Zf),IR4/iTreg-Gata3-ChIP-Seq(GSE20898)/Homer	1e-2	-5.074e+00	0.3120	10.0	0.72%	120.4	0.27%	motif file (matrix)	svg
10	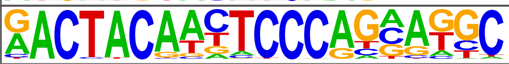	Ronin(THAP)/ES-Thap11-ChIP-Seq(GSE51522)/Homer	1e-2	-4.706e+00	0.3771	69.0	4.95%	1634.9	3.65%	motif file (matrix)	svg
11	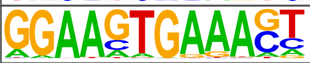	PU.1:IRF8(ETS:IRF)/pDC-Irf8-ChIP-Seq(GSE66899)/Homer	1e-2	-4.630e+00	0.3771	46.0	3.30%	1013.7	2.26%	motif file (matrix)	svg

Homer Known Motif Enrichment Results (FXRa2wt_down)

Homer *de novo* Motif Results
Gene Ontology Enrichment Results
Known Motif Enrichment Results (txt file)
Total Target Sequences = 590, Total Background Sequences = 44570

Rank	Motif	Name	P-value	log P-pvalue	q-value (Benjamini)	# Target Sequences with Motif	% of Targets Sequences with Motif	# Background Sequences with Motif	% of Background Sequences with Motif	Motif File	SVG
1	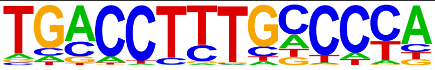	PPARE(NR),DR1/3T3L1-Pparg-ChIP-Seq(GSE13511)/Homer	1e-2	-4.898e+00	1.0000	87.0	14.75%	5058.7	11.35%	motif file (matrix)	svg

Homer Known Motif Enrichment Results (FXRa2L434R_up)

[Homer *de novo* Motif Results](#)
[Gene Ontology Enrichment Results](#)
[Known Motif Enrichment Results (txt file)](#)
Total Target Sequences = 394, Total Background Sequences = 46122

Rank	Motif	Name	P-value	log P-pvalue	q-value (Benjamini)	# Target Sequences with Motif	% of Targets Sequences with Motif	# Background Sequences with Motif	% of Background Sequences with Motif	Motif File	SVG
1	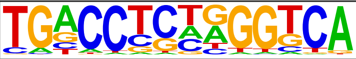	FXRa2(NR)_GSE133700,ER2-motif_15nt(Ramos-Pittol_et_al.)	1e-3	-7.707e+00	0.1875	45.0	11.42%	3108.6	6.74%	motif file (matrix)	svg
2	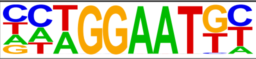	TEAD(TEA)/Fibroblast-PU.1-ChIP-Seq(Unpublished)/Homer	1e-2	-6.873e+00	0.2158	35.0	8.88%	2326.9	5.04%	motif file (matrix)	svg
3	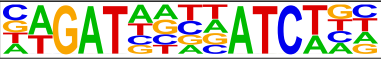	GATA(Zf),IR4/iTreg-Gata3-ChIP-Seq(GSE20898)/Homer	1e-2	-6.549e+00	0.2158	6.0	1.52%	137.9	0.30%	motif file (matrix)	svg
4	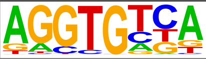	Tbx5(T-box)/HL1-Tbx5.biotin-ChIP-Seq(GSE21529)/Homer	1e-2	-6.081e+00	0.2382	177.0	44.92%	17430.8	37.78%	motif file (matrix)	svg
5	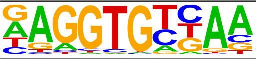	Tbx6(T-box)/ESC-Tbx6-ChIP-Seq(GSE93524)/Homer	1e-2	-6.023e+00	0.2382	65.0	16.50%	5348.8	11.59%	motif file (matrix)	svg
6	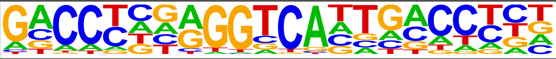	FXRa2(NR)_GSE133700,ER2/IR1-overlapping-motif_22nt(Ramos-Pittol_et_al.)	1e-2	-5.388e+00	0.3177	27.0	6.85%	1820.1	3.94%	motif file (matrix)	svg
7	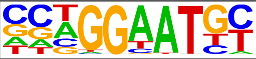	TEAD4(TEA)/Tropoblast-Tead4-ChIP-Seq(GSE37350)/Homer	1e-2	-5.267e+00	0.3177	51.0	12.94%	4120.3	8.93%	motif file (matrix)	svg
8	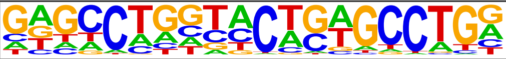	ZNF322(Zf)/HEK293-ZNF322.GFP-ChIP-Seq(GSE58341)/Homer	1e-2	-4.993e+00	0.3538	31.0	7.87%	2247.0	4.87%	motif file (matrix)	svg
9	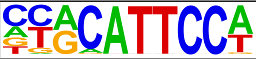	TEAD1(TEAD)/HepG2-TEAD1-ChIP-Seq(Encode)/Homer	1e-2	-4.833e+00	0.3688	52.0	13.20%	4318.2	9.36%	motif file (matrix)	svg

Homer Known Motif Enrichment Results (FXRa2L434R_down)

[Homer *de novo* Motif Results](#)
[Gene Ontology Enrichment Results](#)
[Known Motif Enrichment Results (txt file)](#)
Total Target Sequences = 33, Total Background Sequences = 41986

Rank	Motif	Name	P-value	log P-pvalue	q-value (Benjamini)	# Target Sequences with Motif	% of Targets Sequences with Motif	# Background Sequences with Motif	% of Background Sequences with Motif	Motif File	SVG
1	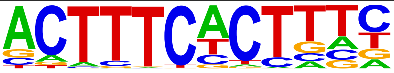	PRDM1(Zf)/Hela-PRDM1-ChIP-Seq(GSE31477)/Homer	1e-2	-5.309e+00	1.0000	7.0	21.21%	2754.3	6.56%	motif file (matrix)	svg
