# Supplementary material for: The farnesoid X receptor activates transcription independently of RXR at non-canonical response elements
Source: Nucleic Acids Res. 2024 Dec 9;53(4):gkae1214. doi: 10.1093/nar/gkae1214 (PMC11879013; doi:10.1093/nar/gkae1214)
Supplement: gkae1214_Supplemental_Files [file gkae1214_supplemental_files.zip › SD8-Alignment of NR sequences.docx]

CLUSTAL O(1.2.4) multiple sequence alignment

RARA ------------------------------------------------------------ 0

RXRB MSWAARPPFLPQRHAAGQCGPVGVRKEMHCGVASRWRRR--------------------- 39

RXRA ------------------------------------MDT--------------------- 3

RXRG ----------------------------------MYGNY--------------------- 5

PPARG --------------------------------MGETLGDSPIDPESDSFTDTLSANISQE 28

PPARA ------------------------------------------------------------ 0

PPARD ------------------------------------------------------------ 0

FXR ----------MVMQFQGLENPIQISPHCSCTPSGFFMEMMSMKPAKGVLTEQVAGPLGQN 50

LXRA ------------------------------------------------------------ 0

LXRB ------------------------------------------------------------ 0

THRA ------------------------------------------------------------ 0

THRB -----------------------------------------MTPN--------------S 5

CAR ------------------------------------------------------------ 0

PXR ------------------------------------------------------------ 0

RARA ------------------------------------------------------------ 0

RXRB -------RPWLDPAAAAAAAVAGGEQQTPEPEPGEAGRDGMGDSGRDSRSPDSSSPNPLP 92

RXRA -------KHFLPLDFSTQ--------------------------------VNSSLTSPTG 24

RXRG -------SHFMK--------------------------------------------FPAG 14

PPARG MTMVDTEMPFWPTNFGI-------------------------SSVDLSVMEDHSHS---- 59

PPARA --MVDTESPLCPLSPLEA-----GDLESPLSEEF-LQEMGNIQEISQSIGEDSSGS---- 48

PPARD -------------------------MEQPQEEAPEVREEEEKEEVAEAEG---------- 25

FXR LE--V------------------------------------------------------- 53

LXRA ------------------------------------------------------------ 0

LXRB -M--S------------------------------------------------------- 2

THRA ------------------------------------------------------------ 0

THRB MT--E------------------------------------------------------- 8

CAR ------------------------------------------------------------ 0

PXR ------------------------------------------------------------ 0

RARA ------------------------MASNSSSCPTPGGGH----LNGYPVPPYAFFFPPML 32

RXRB QGVPPPSPPGPPLPPSTAPSLGG----------SG-----APPPPPMPPPPLGSPFPVIS 137

RXRA RGS----MAAPSLHPSLGPGIG-----------SPGQLHSPISTLSSPINGMGPPFSVIS 69

RXRG YGGSPGHTGSTSMSPSAALSTGKPMDSHPSYTDTPVSAPRTLSAVGTPLNALGSPYRVIT 74

PPARG FDI----------KPFTTVDFS--SISTPHYEDIP-------------FTRTDPVVAD-- 92

PPARA FGF----------T---------------EYQ----------------YLGSCPG-SD-- 64

PPARD --A----------P---------------E-------------------LNGGPQHAL-- 37

FXR -------------------------EPYSQYSNVQ-------------FPQVQPQISS-- 73

LXRA ---------------------------MSLWLGAP-------------VPDIPPDSAV-- 18

LXRB -------------------------SPTTSSLDTP-------------LPGNGPP----- 19

THRA ------------------------------------------------------------ 0

THRB -------------------------NGLTAWD----------------KPKHCPDR-E-- 24

CAR ------------------------------------------------------------ 0

PXR ------------------------------------------------------------ 0

RARA GGLSPPGALTTLQH------------------QLPVSG------YST-PSPATIET---- 63

RXRB SSMGSPGLPPPA---------------------PPGFS------GPV-SSPQINSTVSLP 169

RXRA SPMGPHSMSVPT---------------------TPTLG------FST-GSPQLSSPMN-- 99

RXRG SAMGPPSGALAA---------------------PPGIN------LVA-PPSSQLNVVN-- 104

PPARG YKYDL------------------------------------------KLQEYQSA-IKVE 109

PPARA --------------------------------------------------GSVIT-DTLS 73

PPARD --------------------------------------------------PSSSY-TDLS 46

FXR SSY------------------Y---SNLGFYPQQP-EEWYSPGIYELRRMPAE----TLY 107

LXRA ELWKP-GAQDASSQ----AQGG---SSCILREEAR-MPHSAGGTAGVGLEAAEPT-ALLT 68

LXRB ---QP-GAP------------S---SSPTVKEEGP-EPWPGGPDPDVPGTD-EAS-SACS 57

THRA ---------------------------MEQKPSKVEC--------GS--DPEENS-ARSP 22

THRB HDWKLVGMSEACLHRKSHSERRSTLKNEQSSPHLIQTTWTSSI-FHL--DHDDV-----N 76

CAR ------------------------------------------------------------ 0

PXR ---------------------------MEVR---PKESWNHAD-FVH--CEDTE------ 21

RARA --QSSSSEE------------IVPSPP--SPPPLPRIYKPCFVCQDKSSGYHYGVSACEG 107

RXRB GGGSGPPEDVKPPVLGVRGLH---CPP--PPGGPGAGKRLCAICGDRSSGKHYGVYSCEG 224

RXRA --PVSSSEDIKPP-LGLNGVLKVPAHP--SGNMASFTKHICAICGDRSSGKHYGVYSCEG 154

RXRG --SVSSSEDIKPL-PGLPGIGNM-NYP--STSPGSLVKHICAICGDRSSGKHYGVYSCEG 158

PPARG PASP-PYYSEKT------Q--LYNKPH--EEPSNSLMAIECRVCGDKASGFHYGVHACEG 158

PPARA PASS-PSSVTYP------V---VPGSV--DESPSGALNIECRICGDKASGYHYGVHACEG 121

PPARD RSSS-PPSLLD-------Q---LQMGC--DGASCGSLNMECRVCGDKASGFHYGVHACEG 93

FXR QGET-EVAEM--------P--VTKKPRMGASAGRIKGDELCVVCGDRASGYHYNALTCEG 156

LXRA RAEP-PSEPT--------E--IRPQKRKKGPAPKMLGNELCSVCGDKASGFHYNVLSCEG 117

LXRB TDWV-IPDPE--------E--EPERKRKKGPAPKMLGHELCRVCGDKASGFHYNVLSCEG 106

THRA DGKR-KRKNGQ-------C--SLKTSMSGYIPSYLDKDEQCVVCGDKATGYHYRCITCEG 72

THRB DQSV-SSAQTF-------Q--TEEKKCKGYIPSYLDKDELCVVCGDKATGYHYRCITCEG 126

CAR ------------------------------MASREDELRNCVVCGDQATGYHFNALTCEG 30

PXR ---------------------SVPGKPSVNADEEVGGPQICRVCGDKATGYHFNVMTCEG 60

* :* *:::* *: :***

RARA CKGFFRRSIQKNM--VYTCHRDKNCIINKVTRNRCQYCRLQKCFEVGMSKESVRNDR--- 162

RXRB CKGFFKRTIRKDL--TYSCRDNKDCTVDKRQRNRCQYCRYQKCLATGMKREAVQEER--- 279

RXRA CKGFFKRTVRKDL--TYTCRDNKDCLIDKRQRNRCQYCRYQKCLAMGMKREAVQEER--- 209

RXRG CKGFFKRTIRKDL--IYTCRDNKDCLIDKRQRNRCQYCRYQKCLVMGMKREAVQEER--- 213

PPARG CKGFFRRTIRLKL--IYD-RCDLNCRIHKKSRNKCQYCRFQKCLAVGMSHNAIRFGRMPQ 215

PPARA CKGFFRRTIRLKL--VYD-KCDRSCKIQKKNRNKCQYCRFHKCLSVGMSHNAIRFGRMPR 178

PPARD CKGFFRRTIRMKL--EYE-KCERSCKIQKKNRNKCQYCRFQKCLALGMSHNAIRFGRMPE 150

FXR CKGFFRRSITKNA--VYKCKNGGNCVMDMYMRRKCQECRLRKCKEMGMLAECMYTGLLTE 214

LXRA CKGFFRRSVIKGA--HYICHSGGHCPMDTYMRRKCQECRLRKCRQAGMREECVLSEE--- 172

LXRB CKGFFRRSVVRGGARRYACRGGGTCQMDAFMRRKCQQCRLRKCKEAGMREQCVLSEE--- 163

THRA CKGFFRRTIQKNLHPTYSCKYDSCCVIDKITRNQCQLCRFKKCIAVGMAMDLVLDDSKRV 132

THRB CKGFFRRTIQKNLHPSYSCKYEGKCVIDKVTRNQCQECRFKKCIYVGMATDLVLDDSKRL 186

CAR CKGFFRRTVSKSIGPTCPFA--GSCEVSKTQRRHCPACRLQKCLDAGMRKDMILSAEALA 88

PXR CKGFFRRAMKRNARLRCPFR-KGACEITRKTRRQCQACRLRKCLESGMKKEMIMSDEAVE 119

*****:*:: * : *.:* ** :** ** : :

RARA -NKK-KKEVP-KPECS--ESYT----------------------------------LTPE 183

RXRB -QRG-KDKDG-DGEGA--GGAP----------------------------------EEMP 300

RXRA -QRG-KDRNENEVEST--SSAN----------------------------------EDMP 231

RXRG -QRS-RERAESEAECA--TSGH----------------------------------EDMP 235

PPARG AEKE-KLLAEISS--D---IDQLNPE-------------------------------SAD 238

PPARA SEKA-KLKAEILT-CE---HDIEDSE-------------------------------TAD 202

PPARD AEKR-KLVAGLTA-NE---GSQYNPQ-------------------------------VAD 174

FXR IQCKSKRLRKNVKQHADQTVNE-DSEGRDLRQ-----------VTSTTKSCREKTELTPD 262

LXRA -QIRLKKLKRQEEEQAHATSLP--PRASSPPQ--------------------ILPQLSPE 209

LXRB -QIRKKKIRKQQQESQSQSQSPVGPQGSSSSASGPGASPGGSEAGSQGSGEGEGVQLTAA 222

THRA AKR--KLIEQNRERRR--KEEM-------------------------IRSLQQRPEPTPE 163

THRB AKR--KLIEENREKRR--REEL-------------------------QKSIGHKPEPTDE 217

CAR LRR--AKQAQRRAQQT-----P----------------------------VQ----LSKE 109

PXR ERR--ALIKRKKSERT--GTQP-------------------------LGVQG----LTEE 146

.

RARA VGELIEKVRKAHQE----TFPALCQL-------------------GKYTTNNSSE----- 215

RXRB VDRILEAELAVEQK----SDQGVEGP-------------------GGTGGSG-------- 329

RXRA VERILEAELAVEPK----TETYVEA---------------------NMGLNP-------- 258

RXRG VERILEAELAVEPK----TESYGDM---------------------NME----------- 259

PPARG LRALAKHLYDSYIKSFPLTKAKAR-----AIL-T----------------GKTTDKSPFV 276

PPARA LKSLAKRIYEAYLKNFNMNKVKAR-----VIL-S----------------GKASNNPPFV 240

PPARD LKAFSKHIYNAYLKNFNMTKKKAR-----SIL-T----------------GKASHTAPFV 212

FXR QQTLLHFIMDS---YNKQRMPQE--I------------------------TN-------- 285

LXRA QLGMIEKLVAAQQQCNRRSFSDRLRV------------------------TP-------- 237

LXRB QELMIQQLVAAQLQCNKRSFSDQPKV------------------------TP-------- 250

THRA EWDLIHIATEAHRS-------------------------------TNAQGSHWKQRRKFL 192

THRB EWELIKTVTEAHVA-------------------------------TNAQGSHWKQKRKFL 246

CAR QEELIRTLLGAHTRHMGTMFEQFVQFRPPAHLFIHHQP---------------------- 147

PXR QRMMIRELMDAQMKTFDTTFSHFKNFRLPGVLSSGCELPESLQAPSREEAAKWSQVRKDL 206

: .

RARA -------------------------QRVSLDIDLWDKFSELSTKCIIKTVEFAKQLPGFT 250

RXRB ----------------------------SSPNDPVTNICQAADKQLFTLVEWAKRIPHFS 361

RXRA ----------------------------SSPNDPVTNICQAADKQLFTLVEWAKRIPHFS 290

RXRG ----------------------------NSTNDPVTNICHAADKQLFTLVEWAKRIPHFS 291

PPARG IYDM-NSLMMGEDKIKFKHITPLQEQSKEVAIRIFQGCQFRSVEAVQEITEYAKSIPGFV 335

PPARA IHDM-ETLCMAEKTLVAKLVANGIQ-NKEAEVRIFHCCQCTSVETVTELTEFAKAIPGFA 298

PPARD IHDI-ETLWQAEKGLVWKQLVNGLPPYKEISVHVFYRCQCTTVETVRELTEFAKSIPSFS 271

FXR ---------------------KILKEEF-SAEENFLILTEMATNHVQVLVEFTKKLPGFQ 323

LXRA ----------------W-PMAPDPHSRE-ARQQRFAHFTELAIVSVQEIVDFAKQLPGFL 279

LXRB ----------------W-PLGADPQSRD-ARQQRFAHFTELAIISVQEIVDFAKQVPGFL 292

THRA PDDI---------GQS--PIVSMPDGDK-VDLEAFSEFTKIITPAITRVVDFAKKLPMFS 240

THRB PEDI---------GQA--PIVNAPEGGK-VDLEAFSHFTKIITPAITRVVDFAKKLPMFC 294

CAR -----------------------LPTLA-PVLPLVTHFADINTFMVLQVIKFTKDLPVFR 183

PXR CSLKVSLQLRGEDGSVWNYKPPADSGGK-EIFSLLPHMADMSTYMFKGIISFAKVISYFR 265

. .::* : *

RARA TLTIADQITLLKAACLDILILRICTRYTPEQDTMTFSDGLTLNRT----QMHNAGFGPLT 306

RXRB SLPLDDQVILLRAGWNELLIASFSHRSIDVRDGILLATGLHVHRN----SAHSAGVGAIF 417

RXRA ELPLDDQVILLRAGWNELLIASFSHRSIAVKDGILLATGLHVHRN----SAHSAGVGAIF 346

RXRG DLTLEDQVILLRAGWNELLIASFSHRSVSVQDGILLATGLHVHRS----SAHSAGVGSIF 347

PPARG NLDLNDQVTLLKYGVHEIIYTMLASLMNKDGVLISEGQGF-MTREFL--KSLRKPF-GDF 391

PPARA NLDLNDQVTLLKYGVYEAIFAMLSSVMNKDGMLVAYGNGF-ITREFL--KSLRKPF-CDI 354

PPARD SLFLNDQVTLLKYGVHEAIFAMLASIVNKDGLLVANGSGF-VTREFL--RSLRKPF-SDI 327

FXR TLDHEDQIALLKGSAVEAMFLRSAEIFNKKLPSG----HSDLLEER----IRNSGISDEY 375

LXRA QLSREDQIALLKTSAIEVMLLETSRRYNPGSESITFLKDFSYNRED----FAKAGLQVEF 335

LXRB QLGREDQIALLKASTIEIMLLETARRYNHETECITFLKDFTYSKDD----FHRAGLQVEF 348

THRA ELPCEDQIILLKGCCMEIMSLRAAVRYDPESDTLTLSGEMAVKRE----QLKNGGL-GVV 295

THRB ELPCEDQIILLKGCCMEIMSLRAAVRYDPESETLTLNGEMAVTRG----QLKNGGL-GVV 349

CAR SLPIEDQISLLKGAAVEICHIVLNTTFCLQTQNFLCG-PLRYTIEDGARVSPTVGFQVEF 242

PXR DLPIEDQISLLKGAAFELCQLRFNTVFNAETGTWECG-RLSYCLEDTA-----GGFQQLL 319

* **: **: : .

RARA DLVFA-FANQLLPLEMDDAETGLLSAICLICGDRQDLEQPDRVDMLQEPLLEALKVYVRK 365

RXRB DRVLTELVSKMRDMRMDKTELGCLRAIILFNPDAKGLSNPSEVEVLREKVYASLETYCKQ 477

RXRA DRVLTELVSKMRDMQMDKTELGCLRAIVLFNPDSKGLSNPAEVEALREKVYASLEAYCKH 406

RXRG DRVLTELVSKMKDMQMDKSELGCLRAIVLFNPDAKGLSNPSEVETLREKVYATLEAYTKQ 407

PPARG MEPKFEFAVKFNALELDDSDLAIFIAVIILSGDRPGLLNVKPIEDIQDNLLQALELQLKL 451

PPARA MEPKFDFAMKFNALELDDSDISLFVAAIICCGDRPGLLNVGHIEKMQEGIVHVLRLHLQS 414

PPARD IEPKFEFAVKFNALELDDSDLALFIAAIILCGDRPGLMNVPRVEAIQDTILRALEFHLQA 387

FXR ITPMFSFYKSIGELKMTQEEYALLTAIVILSPDRQYIKDREAVEKLQEPLLDVLQKLCKI 435

LXRA INPIFEFSRAMNELQLNDAEFALLIAISIFSADRPNVQDQLQVERLQHTYVEALHAYVSI 395

LXRB INPIFEFSRAMRRLGLDDAEYALLIAINIFSADRPNVQEPGRVEALQQPYVEALLSYTRI 408

THRA SDAIFELGKSLSAFNLDDTEVALLQAVLLMSTDRSGLLCVDKIEKSQEAYLLAFEHYVNH 355

THRB SDAIFDLGMSLSSFNLDDTEVALLQAVLLMSSDRPGLACVERIEKYQDSFLLAFEHYINY 409

CAR LELLFHFHGTLRKLQLQEPEYVLLAAMALFSPDRPGVTQRDEIDQLQEEMALTLQSYIKG 302

PXR LEPMLKFHYMLKKLQLHEEEYVLMQAISLFSPDRPGVLQHRVVDQLQEQFAITLKSYIEC 379

: : : : . : : * : * : :: :. :

RARA --RRPSRPHMFPKM**L**MKITDLRSISAKGAER----------------VITLKMEIPGSMP 407

RXRB --KYPEQQGRFAKL**L**LRLPALRSIGLKCLEH----------------LFFFKLIGDTPID 519

RXRA --KYPEQPGRFAKL**L**LRLPALRSIGLKCLEH----------------LFFFKLIGDTPID 448

RXRG --KYPEQPGRFAKL**L**LRLPALRSIGLKCLEH----------------LFFFKLIGDTPID 449

PPARG --NHPESSQLFAKL**L**QKMTDLRQIVTEHVQLL---------------QVIKKTETDMSLH 494

PPARA --NHPDDIFLFPKL**L**QKMADLRQLVTEHAQLV---------------QIIKKTESDAALH 457

PPARD --NHPDAQYLFPKL**L**QKMADLRQLVTEHAQMM---------------QRIKKTETETSLH 430

FXR --HQPENPQHFACL**L**GRLTELRTFNHHHAE-----------------MLMSWRVNDHKFT 476

LXRA --HHPHDRLMFPRM**L**MKLVSLRTLSSVHSE-----------------QVFALRLQDKKLP 436

LXRB --KRPQDQLRFPRM**L**MKLVSLRTLSSVHSE-----------------QVFALRLQDKKLP 449

THRA RKHNIPH--FWPKL**L**MKEREVQSSILYKGAAAEGRPGGSLGVHPEGQQLLGMHVVQGPQV 413

THRB RKHHVTH--FWPKL**L**MKVTDLRMIGA----CHAS-------------RFLHMKVECP--- 447

CAR QQRRPRDRFLYAKL**L**GLLAELRSINEAYGY-----------------QIQHIQGLSAM-M 344

PXR NRPQPAHRFLFLKI**M**AMLTELRSINAQHTQ-----------------RLLRIQDIHPFAT 422

: :: ::

RARA PLIQEMLENSEGLDTLSGQPGGGG---------RDGGGLAPPPGSCSPSLS-----PSSN 453

RXRB TFLMEMLEAPHQLA---------------------------------------------- 533

RXRA TFLMEMLEAPHQMT---------------------------------------------- 462

RXRG TFLMEMLETPLQIT---------------------------------------------- 463

PPARG PLLQEIYKDLY------------------------------------------------- 505

PPARA PLLQEIYRDMY------------------------------------------------- 468

PPARD PLLQEIYKDMY------------------------------------------------- 441

FXR PLLCEIWDVQ-------------------------------------------------- 486

LXRA PLLSEIWDVHE------------------------------------------------- 447

LXRB PLLSEIWDVHE------------------------------------------------- 460

THRA RQLEQQLGEAGSLQGPVLQHQSPKSPQQRLLELLHRSGILHARAVCGEDDSSEADSPSSS 473

THRB ----------TELFPPLF------------LEVFED------------------------ 461

CAR PLLQEICS---------------------------------------------------- 352

PXR PLMQELFGITGS------------------------------------------------ 434

RARA RSSPATH--------SP 462

RXRB ----------------- 533

RXRA ----------------- 462

RXRG ----------------- 463

PPARG ----------------- 505

PPARA ----------------- 468

PPARD ----------------- 441

FXR ----------------- 486

LXRA ----------------- 447

LXRB ----------------- 460

THRA EEEPEVCEDLAGNAASP 490

THRB ----------------- 461

CAR ----------------- 352

PXR ----------------- 434

**L434 in FXRa2**
